# Supplementary material for: Theoretical–Computational Modeling of CD Spectra of Aqueous Monosaccharides by Means of Molecular Dynamics Simulations and Perturbed Matrix Method
Source: Molecules. 2023 Apr 20;28(8):3591. doi: 10.3390/molecules28083591 (PMC10144652; doi:10.3390/molecules28083591)

Supplementary Information

for

Theoretical-computational modelling of CD spectra of aqueous monosaccharides by means of Molecular Dynamics simulations and Perturbed Matrix Method

Massimiliano Aschi, Laura Palombi, Andrea Amadei

Outline.
S1. Cartesian coordinates, energies and transition moments for PMM-calculations
S2. Composition of the essential eigenvectors of the covariance matrix
S3. Location of the Representative Conformations on the Essential plane
S4. Spectra of the GG and GT rotamers for Alpha and Beta D-glucose
S5. Eigenvalues of the D-Galactose covariance matrix

S1. Geometries, energies and transition moments for PMM-calculations

The geometries are reported in Angstrom (the first column refers to the atomic number)
The expectation values of the electric dipoles (i,i) are reported in Debye
The transition electric dipoles (i,j) are reported in atomic units
The transition magnetic dipoles (i,j) are reported in atomic units
Ground energy is reported in atomic units
Vertical Excitation energies are reported in eV

D-glucose

Structure A-alpha
Number of atoms : 24

1	-3.80346	-0.68433	0.79396
8	-3.50506	-0.69963	-0.12594
6	-2.59574	0.36188	-0.34719
1	-2.564	0.50143	-1.43175
1	-2.93506	1.29625	0.11314
6	-1.19016	0.05228	0.1451
1	-1.20951	-0.11967	1.23134
6	-0.21995	1.19278	-0.14365
1	-0.27262	1.42733	-1.22137
8	-0.633	2.3102	0.61987
1	0.02668	3.01195	0.5041
6	1.21199	0.79848	0.20164
1	1.2836	0.64004	1.28185
8	2.10196	1.87169	-0.07037
1	2.09256	2.06748	-1.02048
6	1.58616	-0.48143	-0.54171
1	1.57241	-0.29145	-1.62167
8	2.88469	-0.94457	-0.25672
1	3.01402	-0.94757	0.70389
6	0.54548	-1.5705	-0.256291

```
8                 -0.75864  -1.12553  -0.53622
1                  0.6976   -2.43087  -0.9136
8                  0.69072  -1.95525   1.09587
1                 -0.17422  -2.24687   1.42133
Electric dipoles
          0          0 -0.937200010       1.54579997      0.578000009
 0           1   5.92000000E-02 -0.103000000      0.118000001
 0           2  0.153300002      -3.88000011E-02  0.125699997
 0           3  0.129600003     -0.116099998       1.72000006E-02
 0           4   1.09999999E-02   7.93000013E-02  0.147300005
 0           5  -3.46000008E-02  0.146300003     -0.195700005
 0           6  0.224399999      -8.11000019E-02  -5.93000017E-02
 0           7 -0.103699997      0.184000000      0.330199987
 0           8 -0.111100003     -0.195800006       2.58000009E-02
 0           9 -0.102799997     -0.246299997      -4.47999984E-02
 0          10   9.99999978E-03  0.224800006      -7.50999972E-02
 0      11         0.0841     -0.1190     -0.0458      0.0233      0.0046
 0      12        -0.0628      0.1214      0.0803      0.0251      0.0050
 0      13         0.0633      0.1510     -0.3376      0.1408      0.0282
 0      14         0.0412     -0.0736      0.1123      0.0197      0.0040
 0      15        -0.1458     -0.0670     -0.1461      0.0471      0.0096
 0      16        -0.0026      0.1412     -0.0400      0.0216      0.0044
          1          1  -1.68400002      -2.85770011       3.44510007
          2          2   1.65849996       1.52479994      -2.67980003
          3          3  0.548799992       4.08729982      -4.53459978
          4          4  -2.37109995      -4.42729998       5.74720001
          5          5 -0.382600009       2.21860003     -0.981199980
          6          6  -2.12400007      -2.45860004       3.56110001
          7          7  -1.10769999      0.981500030      0.250299990
          8          8   2.17050004       4.28299999      -3.70919991
          9          9  -1.53219998       1.41020000      0.200499997
         10         10  -2.09340000      -1.34010005       2.01440001
11 11   1.8278      5.4269      -0.1257
12 12 -0.9134     -0.4147      -1.1480
13 13 -1.5668     -0.4194       3.6157
14 14 -1.3355      0.0461       1.4263
15 15  0.3037      0.2385      -0.2648
16 16 -2.0189      0.8425       0.7661
Magnetic moments
          1  0.335900009      -4.50999998E-02   1.89999994E-02
          2 -0.283800006     -0.437299997     -0.587800026
          3  -9.71999988E-02 -0.107699998     -0.524999976
          4  0.121100001     -0.294400007     -0.214599997
          5  0.133900002       6.80999979E-02 -0.144199997
          6 -0.314399987       4.54999991E-02  0.674799979
          7 -0.484200001       7.37000033E-02 -0.619000018
          8 -0.856899977      -2.03000009E-02  0.396400005
          9 -0.135499999     -0.373100013       7.25999996E-02
         10 -0.137300000       8.85000005E-02   6.97000027E-02
       11        -0.0488     -0.1443      0.2430
       12         0.3288     -0.3707      0.4956
       13         0.2104      0.0329     -0.3305
```

14	-0.2191	-0.0972	-0.1637
15	-0.0186	0.3791	-0.0576
16	0.2563	-0.2248	0.4416

Ground energy
-686.914978
Vertical Excitation energies
6.69320011
6.76429987
6.96530008
7.15619993
7.31160021
7.43769979
7.63290024
7.65189981
7.70480013
7.92070007
8.0128
8.1414
8.1726
8.2402
8.2766
8.3475

Structure B-alpha
Number of atoms 24

1	-4.01394	-1.04232	-0.49575
8	-3.47016	-0.63667	0.18965
6	-2.59778	0.32913	-0.38461
1	-2.62737	0.29248	-1.4799
1	-2.89391	1.33049	-0.05808
6	-1.18207	0.05892	0.07828
1	-1.21146	-0.12809	1.16158
6	-0.22239	1.22336	-0.19933
1	-0.24232	1.46954	-1.26758
8	-0.64181	2.40231	0.45793
1	-0.52633	2.30186	1.41517
6	1.20074	0.81342	0.19986
1	1.2058	0.62069	1.28181
8	2.13857	1.84799	-0.00174
1	2.5463	1.76127	-0.87572
6	1.58698	-0.4755	-0.52805
1	1.58874	-0.28868	-1.60898
8	2.88874	-0.92037	-0.224
1	2.99357	-0.94877	0.73958
6	0.54768	-1.56668	-0.26484
8	-0.74192	-1.12225	-0.59846
1	0.73014	-2.43393	-0.90649
8	0.65072	-1.92172	1.09485
1	-0.14128	-2.42468	1.34067

Electric dipoles

0	0	-1.50790000	-1.48640001	-0.849600017
0	1	-0.215599999	0.125799999	2.21999995E-02

0 2 -8.79999995E-02 -3.99000011E-02 1.84000004E-02
0 3 0.110699996 -6.47000000E-02 0.146100000
0 4 -5.79000004E-02 1.83000006E-02 -2.50000004E-02
0 5 -1.09999999E-02 4.56000008E-02 0.289000005
0 6 1.42000001E-02 -0.254599988 6.40000030E-02
0 7 -0.120499998 -0.111400001 7.29999989E-02
0 8 5.55999987E-02 -7.28999972E-02 -0.100199997
0 9 4.08999994E-02 0.235100001 0.205300003
0 10 4.63000014E-02 -8.30999985E-02 3.59000005E-02
0 11 -0.0317 -0.2965 0.1478
0 12 0.0399 0.0848 -0.0488
0 13 -0.0250 -0.0758 0.1062
0 14 -0.0847 0.0783 0.2332
0 15 0.3103 -0.0501 -0.0431
0 16 -0.1453 0.3641 -0.1056
1 1 4.07969999 0.908599973 2.61989999
2 2 -1.65489995 -3.31060004 -6.65199995
3 3 -5.65630007 -2.62249994 3.62199998
4 4 -2.75270009 1.47090006 -2.61360002
5 5 -1.28810000 -2.44090009 2.21700001
6 6 -1.54200006 -2.96000000E-02 -0.555100024
7 7 -2.02710009 -1.75820005 -1.42739999
8 8 7.01000020E-02 3.89619994 -3.20869994
9 9 -1.44360006 -3.72539997 -2.31200004
10 10 -2.11089993 1.24839997 -4.16090012
11 11 -1.8993 0.6614 0.5506
12 12 5.6871 -0.2600 1.3221
13 13 -2.6187 -0.9086 -0.8635
14 14 -0.7052 -0.7120 0.5859
15 15 -2.2486 -0.7730 -3.1810
16 16 -1.4871 -2.5930 1.2248
Magnetic moments
1 0.183300003 -4.60000010E-03 0.654999971
2 -3.84000018E-02 0.328700006 -0.160500005
3 -1.00999996E-02 0.407499999 0.185900003
4 -0.132699996 0.184499994 0.734899998
5 0.189600006 -0.259700000 2.49999994E-03
6 -0.278499991 0.139599994 7.78999999E-02
7 -0.215900004 9.89999995E-03 -0.899900019
8 0.668299973 -4.47999984E-02 3.84999998E-02
9 -0.257299989 -8.00000038E-03 2.03000009E-02
10 -0.411900014 -7.00000003E-02 -1.83000006E-02
11 -0.4018 -0.3612 0.3345
12 -0.0916 -0.1752 0.0980
13 0.0896 0.0061 0.0937
14 0.0070 0.2742 -0.2740
15 -0.0861 -0.2237 0.5213
16 0.0806 0.0800 0.2118
Ground energy
-686.913208
Excitation energies
6.72840023

6.83020020
7.02829981
7.06829977
7.28310013
7.38749981
7.52530003
7.68779993
7.78020000
7.87589979
7.8858
7.9871
8.1295
8.1473
8.2316
8.3065

Structure C-alpha
Number of atom: 24

1	-3.14896	-1.35098	-0.75953
8	-3.45166	-0.6781	-0.13282
6	-2.60073	0.44226	-0.26129
1	-2.56953	0.79173	-1.30296
1	-3.02209	1.23979	0.35245
6	-1.18664	0.12432	0.20357
1	-1.19307	-0.09463	1.27985
6	-0.18803	1.23037	-0.11646
1	-0.27906	1.50135	-1.17279
8	-0.49571	2.42547	0.5959
1	-0.32798	2.28375	1.54105
6	1.25591	0.80529	0.14341
1	1.42317	0.69184	1.22546
8	2.14702	1.75617	-0.40423
1	2.05481	2.58668	0.08468
6	1.55771	-0.5298	-0.51955
1	1.55385	-0.37069	-1.60779
8	2.8334	-0.94893	-0.08582
1	2.96419	-1.88495	-0.29434
6	0.47033	-1.56459	-0.21165
8	-0.80735	-1.05866	-0.51846
1	0.57682	-2.43759	-0.86869
8	0.5701	-1.94585	1.13506
1	-0.24992	-2.40417	1.37741

Electric dipoles

0	0	-0.589399993	-1.64110005	0.337700009
0	1	-1.54999997E-02	8.51000026E-02	7.40000000E-03
0	2	-0.100000001	3.75000015E-02	-1.85000002E-02
0	3	-0.217999995	8.34000036E-02	-0.105200000
0	4	7.06999972E-02	-0.176899999	0.141399994
0	5	0.241500005	4.47999984E-02	-0.143199995
0	6	6.94999993E-02	0.155200005	0.180099994
0	7	9.74000022E-02	0.216299996	-0.202999994
0	8	-1.66999996E-02	7.24999979E-02	0.396699995

0 9 7.49999983E-03 0.130500004 0.154200003
0 10 -8.89999978E-03 -0.173999995 -1.24000004E-02
0 11 0.0503 -0.1141 -0.1633 0.0422 0.0082
0 12 0.0216 0.0561 -0.0618 0.0074 0.0015
0 13 -0.0286 0.1315 0.0483 0.0204 0.0041
0 14 0.1495 -0.1135 -0.2699 0.1081 0.0215
0 15 -0.1825 0.0940 0.0196 0.0425 0.0086
0 16 -0.0221 -0.2781 0.2207 0.1265 0.0256
1 1 -1.36960006 -5.54379988 -4.78889990
2 2 -2.90420008 4.73280001 2.68700004
3 3 0.801500022 2.68659997 2.08629990
4 4 2.21530008 3.16149998 -1.75870001
5 5 -1.04939997 -3.90050006 -2.15969992
6 6 1.88720000 -3.07000000E-02 1.29400003
7 7 -0.657000005 -1.40840006 -0.983699977
8 8 -1.27139997 3.10000009E-03 -3.63369989
9 9 -1.94430006 1.69309998 0.669900000
10 10 -0.210099995 -0.219099998 0.274399996
11 11 -1.8222 0.3580 0.9934
12 12 -0.9045 -3.0999 -3.5985
13 13 0.7624 -2.0270 -1.3049
14 14 -2.0858 -1.8855 0.5922
15 15 5.3702 1.9463 -0.2296
16 16 0.1317 1.6093 0.9862
Magnetic moments
1 0.106600001 -0.370000005 0.128600001
2 0.310600013 0.416999996 0.410699993
3 -0.330599993 0.445499986 0.355599999
4 -3.04000005E-02 0.150500000 5.27999997E-02
5 -0.172199994 -2.19999999E-02 0.657700002
6 0.471899986 -0.141700000 0.324200004
7 0.180000007 3.57999988E-02 0.180000007
8 -0.448900014 -0.160300002 -3.95999998E-02
9 -1.72000006E-02 0.445499986 0.225099996
10 -6.94999993E-02 0.290100008 7.55999982E-02
11 -0.3414 -0.5560 0.2965
12 0.5719 -0.1556 -0.2448
13 -0.3802 0.4980 0.1997
14 -0.0917 0.1238 0.0885
15 0.2921 0.3815 -0.0576
16 0.0072 -0.0622 0.0276
Ground energy
-686.914062
Excitation energies
6.62169981
6.81050014
7.03739977
7.20149994
7.25939989
7.54680014
7.61579990
7.70030022

7.85920000
7.88560009
7.9077
8.0372
8.0996
8.1300
8.2291
8.2686

Structure D-alpha
Number of atomd: 24

1	-4.3575	-0.44373	-0.24175
8	-3.45034	-0.69071	-0.01838
6	-2.57973	0.37225	-0.36829
1	-2.54622	0.50205	-1.45978
1	-2.89023	1.31645	0.09446
6	-1.19071	0.03674	0.12719
1	-1.23305	-0.1784	1.20133
6	-0.21397	1.19316	-0.12763
1	-0.26717	1.45769	-1.19389
8	-0.62197	2.34707	0.60522
1	0.0395	2.57285	1.27504
6	1.21103	0.78046	0.19686
1	1.31205	0.60871	1.27694
8	2.17005	1.76831	-0.18711
1	1.72708	2.58653	-0.45437
6	1.55839	-0.51093	-0.53119
1	1.48694	-0.32529	-1.61228
8	2.84613	-0.97126	-0.19762
1	3.47485	-0.26412	-0.41002
6	0.52546	-1.58204	-0.19571
8	-0.74985	-1.12134	-0.578
1	0.70288	-2.47719	-0.79744
8	0.5943	-1.86895	1.174
1	0.38142	-2.79837	1.32951

Electric dipoles

0	0	-0.865000010	1.73490000	-0.853299975		
0	1	3.89999989E-03	3.68999988E-02	0.118000001		
0	2	6.12000003E-02	-9.00000036E-02	0.135800004		
0	3	-5.44999987E-02	-2.00999994E-02	0.118600003		
0	4	5.00000007E-02	0.161899999	0.121399999		
0	5	7.82999992E-02	-0.117299996	3.64000015E-02		
0	6	-0.105200000	3.33000012E-02	-3.20999995E-02		
0	7	-0.247400001	2.47000009E-02	-9.20000020E-03		
0	8	4.60000010E-03	-1.84000004E-02	-0.316300005		
0	9	-0.162799999	-0.119800001	0.176699996		
0	10	1.96000002E-02	5.53999990E-02	0.229000002		
0	11	0.1329	-0.0995	0.3232	0.1321	0.0258
0	12	-0.0032	-0.1971	-0.2498	0.1013	0.0199
0	13	-0.0162	-0.1276	0.2716	0.0903	0.0178
0	14	-0.1047	-0.0996	0.1709	0.0501	0.0100
0	15	-0.2021	-0.0943	0.2591	0.1169	0.0235

0	16	0.0825	-0.0494	-0.1475	0.0310	0.0063
	1	1	5.96059990	0.484699994	0.634100020	
	2	2	-3.01929998	-4.56850004	-1.55170000	
	3	3	-4.44000006	-1.71519995	-0.831600010	
	4	4	-2.21269989	0.906300008	0.133900002	
	5	5	-2.41790009	5.34119987	0.750199974	
	6	6	-1.40020001	1.07319999	-1.00660002	
	7	7	-0.415499985	8.52770042	-2.47900009	
	8	8	0.808600008	-2.75939989	-0.574899971	
	9	9	6.29449987	-7.89999962E-03	0.661000013	
	10	10	1.28400004	1.23549998	-2.00029993	
11	11	-0.8846	-0.0528	0.7072		
12	12	-3.4524	-2.7081	-2.6006		
13	13	-2.9906	-1.9849	0.4471		
14	14	-6.6475	-4.7816	-4.0475		
15	15	-2.8149	3.9473	0.6003		
16	16	-3.3277	5.4574	-1.6607		

Magnetic moments

1	0.298400015	-0.576499999	0.346399993
2	-0.694800019	0.106399998	-0.213799998
3	-8.37000012E-02	0.282700002	-0.351599991
4	4.17999998E-02	0.385800004	-0.166999996
5	0.235200003	-0.581200004	-0.184200004
6	3.28000002E-02	-0.426699996	0.236399993
7	6.92000017E-02	0.325800002	0.321000010
8	3.13999988E-02	-0.545700014	3.42000015E-02
9	-0.291399986	0.331499994	-0.603299975
10	-0.306699991	0.322299987	-0.180000007
11	0.1023	-0.1205	0.2647
12	-0.0315	0.2897	-0.2990
13	-0.2106	-0.5472	-0.2614
14	0.3603	-0.3068	-0.1255
15	0.1144	-0.0132	0.0575
16	0.2787	0.0717	0.0623

Ground energy
-686.912048
Excitation energy
6.60580015
6.79740000
7.06059980
7.27920008
7.37309980
7.48579979
7.57060003
7.75719976
7.82789993
7.94430017
7.9815
8.0079
8.0493
8.1862
8.2158

8.2628

Structure E-alpha
Number of atoms: 24

1	3.0975	0.83995	-0.32794
8	3.48538	-0.03114	-0.13828
6	2.50246	-0.86652	0.43546
1	2.76535	-1.90037	0.19594
1	2.49892	-0.76821	1.53206
6	1.09247	-0.58381	-0.07451
1	1.05126	-0.76614	-1.15509
6	0.6083	0.83812	0.19797
1	0.70185	1.03547	1.27894
8	1.4166	1.75464	-0.5222
1	0.96874	2.61681	-0.51158
6	-0.85401	0.99602	-0.20469
1	-0.94866	0.86356	-1.28591
8	-1.25423	2.34194	0.05015
1	-1.66883	2.41531	0.92203
6	-1.71045	-0.05369	0.49174
1	-1.6563	0.09322	1.58121
8	-3.05306	-0.03981	0.05049
1	-3.59727	0.49926	0.63823
6	-1.12295	-1.44337	0.21559
8	0.23013	-1.49456	0.61268
1	-1.62813	-2.19334	0.83167
8	-1.28291	-1.71006	-1.14627
1	-1.01467	-2.62419	-1.32156

Electric dipoles

0	0	-3.58360004	0.235200003	3.08489990
0	1	-1.20999999E-02	-0.170000002	8.16999972E-02
0	2	-7.68000036E-02	-9.47000012E-02	-0.108900003
0	3	8.87999982E-02	5.62999994E-02	5.68000004E-02
0	4	0.151299998	7.94000030E-02	0.205500007
0	5	7.24000037E-02	-0.139300004	5.92000000E-02
0	6	-5.00999987E-02	-1.61000006E-02	0.160899997
0	7	1.65999997E-02	1.13000004E-02	9.13999975E-02
0	8	-0.108300000	0.112099998	0.247899994
0	9	-0.274599999	-7.85000026E-02	-4.99999989E-03
0	10	-0.146699995	-0.102200001	-5.33000007E-02
0	11	-0.0969	-0.0630	0.3581
0	12	0.0801	-0.1490	0.0375
0	13	-0.1436	-0.2226	0.0926
0	14	0.0903	0.0746	0.1253
0	15	-0.0306	0.0411	-0.1202
0	16	0.0555	-0.0100	0.0014
1	1	1.43850005	-3.24099994	-1.83910000
2	2	1.47440004	-2.84739995	-1.45350003
3	3	-2.02679992	-4.14150000	1.37979996
4	4	-2.27430010	5.95489979	2.88479996
5	5	-2.15980005	-3.76239991	1.20980000
6	6	9.44340038	-3.41240001	-1.33560002

7 7 1.78520000 5.96099997 3.15490007
8 8 0.467799991 -0.366800010 -0.128600001
9 9 -1.11660004 -1.13810003 1.00160003
10 10 -0.173099995 1.55390000 -2.30000005E-03
11 11 4.2199 0.2617 1.0464
12 12 -1.3481 1.8788 3.0193
13 13 -0.3294 -1.7056 -0.2396
14 14 -0.2759 0.9669 -0.2261
15 15 -0.2903 -1.4227 -0.1269
16 16 1.5326 0.7414 1.2596
Magnetic moments
1 0.108599998 -0.315200001 -0.227400005
2 0.182699993 0.243399993 -0.588699996
3 -0.309700012 0.455799997 -0.182500005
4 -7.72999972E-02 0.131500006 -0.318500012
5 -0.535200000 0.131799996 0.194999993
6 -7.80000016E-02 0.474099994 -0.159600005
7 0.203500003 0.243100002 -0.247400001
8 0.320600003 0.446000010 0.363400012
9 0.254799992 -0.516900003 -0.301099986
10 -0.316700011 1.18000004E-02 -0.250200003
11 0.0993 0.4600 0.0597
12 -0.1306 0.2516 0.0425
13 -0.2767 -0.1520 -0.5021
14 0.0327 0.3211 0.0581
15 0.0635 0.0086 0.4691
16 -0.3639 0.0082 0.1538
Ground energy
-686.916565
Excitation energies
6.39370012
6.79099989
7.10580015
7.30039978
7.38059998
7.49919987
7.59200001
7.61269999
7.71509981
7.76999998
7.8000
7.9052
7.9928
8.0773
8.1230
8.2354

Structure F-alpha
Number of atoms : 24
1 -3.18635 0.87255 0.00315
8 -3.47059 -0.04599 0.12666
6 -2.4975 -0.91225 -0.40955

1	-2.76508	-1.92523	-0.10123
1	-2.50486	-0.88609	-1.5111
6	-1.07819	-0.60906	0.06861
1	-1.0133	-0.83453	1.13983
6	-0.62094	0.83752	-0.14149
1	-0.73752	1.06105	-1.21814
8	-1.43333	1.72466	0.60935
1	-1.21051	2.6282	0.3374
6	0.85306	1.02168	0.21407
1	0.98169	0.93033	1.29519
8	1.25585	2.35185	-0.10028
1	1.36809	2.43603	-1.05984
6	1.713	-0.03046	-0.48062
1	1.67205	0.13169	-1.56825
8	3.04973	-0.00341	-0.0203
1	3.62378	0.40402	-0.68042
6	1.13285	-1.42651	-0.22581
8	-0.20791	-1.47503	-0.65621
1	1.66137	-2.16394	-0.83576
8	1.2554	-1.71469	1.13815
1	1.50379	-2.63864	1.26892

Electric dipoles

0	0	3.29229999	-3.88999991E-02	-4.03560019		
0	1	-2.52999999E-02	0.103000000	8.87999982E-02		
0	2	3.24999988E-02	0.167999998	4.10000002E-03		
0	3	-0.232500002	6.67999983E-02	-2.64999997E-02		
0	4	0.112700000	0.164499998	1.11999996E-02		
0	5	3.80000006E-03	2.58000009E-02	-0.188099995		
0	6	0.230299994	-0.224600002	3.53000015E-02		
0	7	7.24999979E-02	0.118900001	0.149900004		
0	8	0.294099987	8.22999999E-02	-0.255800009		
0	9	-0.118299998	0.155300006	3.10999993E-02		
0	10	-1.46000003E-02	1.65999997E-02	4.39000018E-02		
0	11	0.2314	-0.0908	0.1681	0.0900	0.0172
0	12	-0.0439	0.0359	-0.0601	0.0068	0.0013
0	13	-0.0299	0.1054	-0.3104	0.1084	0.0210
0	14	0.1404	0.1245	0.2055	0.0775	0.0152
0	15	0.0278	-0.1661	-0.2172	0.0755	0.0149
0	16	0.0756	-0.0979	0.0429	0.0171	0.0034
1	1	-0.672599971	-2.99119997	1.46500003		
2	2	0.883700013	-4.47310019	0.322800010		
3	3	2.35630012	-4.66750002	-0.777400017		
4	4	1.20630002	-2.97839999	-0.510299981		
5	5	0.753700018	5.42589998	-2.97219992		
6	6	1.87950003	-4.40089989	0.458099991		
7	7	-2.00200009	3.53340006	-2.25370002		
8	8	-1.98559999	-0.352400005	-0.824000001		
9	9	-1.03279996	0.908200026	-1.45930004		
10	10	0.150999993	3.01869988	-3.31769991		
11	11	-2.0892	-0.6333	-1.0829		
12	12	0.4341	0.7993	-2.5828		
13	13	-1.2531	1.4122	-1.4468		

14 14   -1.2899   -0.9838   -0.3641
15 15   -1.3867   0.4054   -1.6513
16 16   -0.3609   -2.6547   -0.1448

Magnetic moments

1 0.196999997   0.353399992   -0.153300002
2 -2.65999995E-02 -0.307900012   -0.383500010
3 0.342599988   0.325700015   -0.427300006
4 -0.218700007   -0.113499999   0.577099979
5   7.95999989E-02   7.60000013E-03   8.59000012E-02
6 0.439799994   -0.305000007   0.680999994
7 0.137700006   -0.465499997   -0.551199973
8   6.23000003E-02 0.457899988   -0.247700006
9 0.166099995   0.137199998   -0.100100003
10 0.182999998   8.03999975E-02 0.140900001
11   -0.0376   -0.2687   0.1196
12   -0.1950   0.1822   -0.2335
13   -0.1124   0.0317   -0.0104
14   -0.4148   -0.0159   0.0679
15   -0.4225   0.4701   -0.0761
16   0.3530   0.0312   -0.1010

Ground energy

-686.912170

Excitation energy

6.33839989
6.64239979
6.89659977
7.16510010
7.27829981
7.37559986
7.44759989
7.51889992
7.63420010
7.71939993
7.7913
7.8220
7.9221
8.0096
8.0450
8.1568

Structure G-alpha

Number of atoms: 24

1	-3.4746	0.73465	-0.25684
8	-3.45899	-0.11145	0.21349
6	-2.47284	-0.94687	-0.36507
1	-2.68798	-1.96631	-0.03833
1	-2.52549	-0.92643	-1.46185
6	-1.05625	-0.57837	0.07307
1	-0.96773	-0.76496	1.15404
6	-0.64943	0.87462	-0.20681
1	-0.74908	1.0794	-1.28123
8	-1.48457	1.80987	0.4546

1	-1.89098	1.39214	1.22938
6	0.81597	1.0731	0.19639
1	0.88438	0.94639	1.28521
8	1.25825	2.38538	-0.07832
1	1.82767	2.38666	-0.86061
6	1.69325	0.02093	-0.48715
1	1.68701	0.21794	-1.56705
8	3.04611	0.1191	-0.09771
1	3.12558	-0.26917	0.78844
6	1.15031	-1.39665	-0.29031
8	-0.19781	-1.47482	-0.6286
1	1.66429	-2.08499	-0.96681
8	1.3565	-1.79951	1.05545
1	2.06492	-2.45446	1.10359

Electric dipoles

0	0	1.72950006	-3.22770000	-0.978699982		
0	1	-0.203700006	-2.80000009E-02	-0.103100002		
0	2	0.159899995	-0.143999994	-0.143299997		
0	3	8.30999985E-02	5.27999997E-02	0.316500008		
0	4	4.52999994E-02	0.179600000	-0.159199998		
0	5	-0.233500004	0.249300003	-6.03000000E-02		
0	6	0.166600004	0.171499997	6.78000003E-02		
0	7	0.224500000	-2.72000004E-02	-4.58000004E-02		
0	8	-4.98999991E-02	7.19999969E-02	6.09999988E-03		
0	9	1.53999999E-02	-6.99999975E-04	0.100800000		
0	10	9.82000008E-02	1.76999997E-02	-6.67999983E-02		
0	11	-0.0875	0.0078	0.1240	0.0231	0.0045
0	12	0.3148	0.1491	0.1125	0.1340	0.0262
0	13	0.0368	0.2578	0.2726	0.1421	0.0284
0	14	0.0087	0.1207	0.1688	0.0431	0.0086
0	15	0.0842	0.3533	-0.2982	0.2208	0.0445
0	16	0.0311	-0.1258	-0.1147	0.0299	0.0061
1	1	5.28230000	-4.64440012	-1.25440001		
2	2	-2.87140012	2.47280002	-2.81480002		
3	3	-2.08990002	-3.90680003	3.75029993		
4	4	-1.21169996	-0.293500006	-1.82400000		
5	5	-2.79480004	2.45840001	0.186800003		
6	6	-2.88150001	0.460099995	-9.48999971E-02		
7	7	0.184000000	-1.08990002	0.580200016		
8	8	3.72989988	-4.08269978	1.25849998		
9	9	-0.709200025	-2.45239997	1.24129999		
10	10	-9.30000003E-03	-0.383300006	-2.34859991		
11	11	3.4263	-2.4035	-3.0387	Tot=	5.1721
12	12	2.6380	-0.3815	-1.5272	Tot=	3.0719
13	13	2.8893	-0.1995	-0.0857	Tot=	2.8975
14	14	-0.0622	0.4467	-0.4042	Tot=	0.6057
15	15	0.8602	-1.0197	2.5304	Tot=	2.8606
16	16	-0.5762	-1.2728	2.8810	Tot=	3.2019

Magnetic moment

1	3.99999990E-04	0.762899995	-0.488999993
2	9.36999992E-02	-0.205300003	0.294400007
3	-0.265399992	4.76000011E-02	0.494899988

4 -2.67999992E-02 8.25999975E-02 0.256700009
5 6.93000033E-02 -3.50000011E-03 -0.214699998
6 0.644500017 6.71000034E-02 -0.765600026
7 -1.00000005E-03 0.185699999 -0.301299989
8 9.10999998E-02 -0.213799998 -0.192399994
9 -0.290600002 -4.89999987E-02 -3.02000009E-02
10 -0.345400006 0.324600011 -0.185699999
11 -0.1996 -0.1748 -0.3405
12 -0.2165 -0.1814 0.4792
13 -0.1544 -0.1977 0.1645
14 -0.1916 0.0544 -0.1134
15 -0.1819 0.2069 -0.3250
16 -0.4788 0.5269 -0.2067

Ground energy
-686.908936

Excitation energies
6.71420002
6.83790016
6.93120003
7.08479977
7.26609993
7.42189980
7.46799994
7.54969978
7.67500019
7.88180017
7.9106
7.9850
8.1596
8.1837
8.2197
8.2599

Structure H-alpha

Number of atoms: 24

1	-4.11961	0.27249	-0.57445
8	-3.43274	0.05574	0.06972
6	-2.45555	-0.78616	-0.54201
1	-2.73227	-1.83928	-0.41897
1	-2.36649	-0.57551	-1.61474
6	-1.10439	-0.54403	0.11839
1	-1.18516	-0.69329	1.20185
6	-0.59478	0.88826	-0.11559
1	-0.7728	1.17493	-1.16621
8	-1.25527	1.76653	0.77043
1	-2.19412	1.79408	0.52772
6	0.90023	0.99504	0.17677
1	1.03845	0.87364	1.25529
8	1.43393	2.27214	-0.11361
1	1.40039	2.43184	-1.06927
6	1.65466	-0.10713	-0.56049
1	1.47169	-0.01284	-1.64086

```
8          3.05004  -0.04142  -0.37755
1          3.25141   0.54011   0.36986
6          1.1072   -1.47332  -0.15319
8         -0.26699  -1.54252  -0.47217
1          1.57273  -2.27179  -0.73863
8          1.33374  -1.66317   1.22027
1          0.6899   -2.30875   1.54856
Electric dipoles
   0     0  -3.79780006     -1.36199999     -2.83240008
 0     1  0.115199998     0.185100004      5.02000004E-02
 0     2  -2.51000002E-02   1.99999996E-02 -0.122400001
 0     3  0.179700002     -0.101999998     -3.18000019E-02
 0     4   8.83999988E-02 -0.151800007      0.103100002
 0     5  -1.59000009E-02  0.109899998      -1.35000004E-02
 0     6   3.59999994E-03   8.78999978E-02 -0.354200006
 0     7  -1.26999998E-02   5.49999997E-03  -7.20999986E-02
 0     8   1.09999999E-02  0.121799998       8.65999982E-02
 0     9   5.11000007E-02 -0.180800006      -2.51000002E-02
 0    10 -0.122800000      -4.05000001E-02  0.106399998
 0   11     -0.0290    -0.2398    -0.1330     0.0760     0.0146
 0   12      0.0388    -0.1830     0.0096     0.0351     0.0068
 0   13      0.1057     0.2395     0.0399     0.0701     0.0137
 0   14     -0.2117     0.2019     0.0321     0.0866     0.0171
 0   15     -0.0135     0.0721    -0.0154     0.0056     0.0011
 0   16     -0.0175     0.4209    -0.0718     0.1826     0.0369
   1     1   2.65330005     -2.77390003      0.934300005
   2     2 -0.148599997     -4.23229980      2.01220012
   3     3  -1.91410005     -3.89619994      1.72800004
   4     4  -2.80749989      3.58410001     -4.95620012
   5     5  -3.88989997     -3.60249996     -1.06480002
   6     6  -3.77060008     -2.24020004    -0.656099975
   7     7 -0.518800020      0.768999994     -1.93990004
   8     8  -3.04080009      1.43040001     -1.82299995
   9     9   2.05539989     -1.91009998    -0.475600004
  10    10 -0.496499985     -1.59340000    -0.939700007
11 11   -3.6080     -1.3731    -0.2397  Tot=         3.8679
12 12    3.6290      0.6710    -1.1149  Tot=         3.8553
13 13   -0.3797      4.5867    -4.9134  Tot=         6.7323
14 14   -3.3302     -0.2530     0.0727  Tot=         3.3406
15 15   -0.9201      2.1514    -2.1538  Tot=         3.1802
16 16    0.2729      1.6530    -1.4715  Tot=         2.2298
Magnetic moments
   1  0.335999995     -6.78000003E-02  0.716600001
   2 -0.277200013     -9.45999995E-02 -0.128800005
   3   5.93999997E-02  0.305599988      0.617100000
   4   4.34999987E-02 -0.281500012     -0.626500010
   5  0.289099991     -0.282400012     -0.350499988
   6 -0.213799998     -0.213799998      7.60000013E-03
   7  0.412699997      0.220799997     -5.66999987E-02
   8 -0.548600018      9.10999998E-02  0.165700004
   9 -0.232800007     -0.229200006      8.06000009E-02
  10 -0.462500006     -0.361299992     -0.506399989
```

11	0.3370	-0.0655	0.2026
12	0.0100	0.3814	0.0046
13	0.3161	0.3854	0.3071
14	0.0839	0.1610	0.0537
15	0.0185	-0.2448	0.0191
16	-0.0608	0.3117	0.4323

Energy ground
-686.912109
Excitation energy
6.66590023
6.95709991
7.01370001
7.04230022
7.25120020
7.33300018
7.56610012
7.61250019
7.71820021
7.79879999
7.8311
7.9417
8.0016
8.0732
8.1998
8.2393

Structure I-alpha
Number of atoms: 24

1	-2.32995	-0.89257	-1.58911
8	-2.97086	-0.30868	-1.15419
6	-2.67738	-0.32141	0.23042
1	-3.16245	0.55138	0.67212
1	-3.08242	-1.22853	0.70002
6	-1.18357	-0.26307	0.50112
1	-1.00178	-0.3782	1.57616
6	-0.53106	1.03264	0.02117
1	-0.78764	1.19823	-1.0347
8	-1.03087	2.09141	0.81303
1	-0.44382	2.85153	0.67521
6	0.9798	0.91896	0.15664
1	1.23715	0.79603	1.2148
8	1.55229	2.12826	-0.34228
1	2.32739	2.35486	0.18862
6	1.48076	-0.28835	-0.62034
1	1.201	-0.17184	-1.67508
8	2.88045	-0.45309	-0.49652
1	3.31278	0.04074	-1.20613
6	0.77386	-1.53922	-0.10404
8	-0.62533	-1.37532	-0.21657
1	1.00947	-2.4046	-0.73032
8	1.16625	-1.74777	1.22137
1	0.8904	-2.63283	1.49989

Electric dipoles

0	0	3.06620002	-0.981700003	0.136700004
0	1	7.69999996E-02	0.105700001	-4.01999988E-02
0	2	3.37000005E-02	0.208000004	0.201100007
0	3	0.201299995	5.97999990E-02	3.53999995E-02
0	4	-0.247199997	9.10000037E-03	-4.19999985E-03
0	5	0.228000000	-0.206499994	5.70000000E-02
0	6	-1.04000000E-02	-0.117200002	7.85999969E-02
0	7	-0.266400009	-0.235300004	0.129800007
0	8	5.53000011E-02	-0.170499995	7.59999976E-02
0	9	8.26999992E-02	-0.257900000	0.166600004
0	10	-4.08000015E-02	-0.104900002	0.101899996

0	11	0.0153	0.0292	-0.1363	0.0197	0.0039
0	12	0.0454	-0.0841	-0.0458	0.0112	0.0022
0	13	-0.0015	0.0818	0.0397	0.0083	0.0016
0	14	0.0941	-0.0667	-0.4298	0.1981	0.0397
0	15	0.0152	-0.0009	0.1137	0.0132	0.0026
0	16	0.1158	0.0066	-0.4654	0.2301	0.0468

1	1	-2.44650006	-4.40740013	1.35609996
2	2	-2.43999995E-02	-2.17829990	1.99689996
3	3	1.85350001	1.92279994	3.66479993
4	4	2.62000002E-02	-2.98650002	-0.852699995
5	5	1.82760000	3.63229990	-1.57459998
6	6	2.26430011	-0.485100001	-0.616800010
7	7	2.46000004	4.65170002	-3.07870007
8	8	2.63070011	2.38459992	-2.48160005
9	9	2.20289993	7.44080019	-0.136199996
10	10	-6.25409985	-5.03009987	1.38999999

11	11	-0.7238	-0.8604	-1.4984	Tot=	1.8733
12	12	0.3655	1.1233	-0.0536	Tot=	1.1825
13	13	-3.0533	0.3192	1.9236	Tot=	3.6228
14	14	0.8276	-1.6717	2.5488	Tot=	3.1584
15	15	-7.0035	-7.2021	1.5173	Tot=	10.1598
16	16	2.5373	4.1377	-1.7290	Tot=	5.1525

Magnetic moments

1	0.339899987	0.279599994	-0.124899998
2	-0.393700004	3.72000001E-02	-0.473300010
3	-0.484200001	0.252900004	0.277500004
4	0.181299999	0.244499996	-0.687099993
5	0.105099998	-0.359600008	-9.29000005E-02
6	-0.416700006	5.16000018E-02	-0.620500028
7	0.500299990	-0.135399997	6.49999976E-02
8	1.57999992E-02	-0.670000017	3.81999984E-02
9	0.437400013	-0.208399996	-0.247899994
10	-0.210899994	-0.370400012	0.179499999
11	0.3706	0.2117	0.2085
12	0.0539	-0.3313	0.0115
13	0.0836	0.0078	-0.3541
14	0.4182	-0.3416	0.5442
15	-0.2055	-0.1683	-0.1494
16	0.6797	-0.0937	0.0563

Energy ground

-686.923096
Excitation energy
6.56209993
6.96950006
7.12620020
7.25600004
7.33290005
7.60230017
7.65969992
7.75500011
7.86969995
7.93230009
8.0088
8.0386
8.0856
8.1867
8.1999
8.3049

Structure L-alpha
Number of atoms 24

1	-2.40745	0.02159	-1.60495
8	-3.12281	-0.28364	-1.03278
6	-2.64584	-0.51667	0.28181
1	-3.13469	0.18836	0.96296
1	-2.92862	-1.53534	0.56778
6	-1.14465	-0.38099	0.46438
1	-0.92426	-0.52171	1.52997
6	-0.56506	0.96014	0.01719
1	-0.79487	1.13678	-1.04433
8	-1.15133	1.97372	0.81245
1	-0.90629	2.8333	0.44024
6	0.95778	0.95652	0.1624
1	1.20222	0.81347	1.22352
8	1.49591	2.18924	-0.29841
1	1.59587	2.79819	0.44499
6	1.54592	-0.19103	-0.64941
1	1.31672	-0.03612	-1.70818
8	2.94577	-0.30768	-0.56807
1	3.23557	-0.09511	0.33054
6	0.87335	-1.49474	-0.19997
8	-0.52846	-1.42105	-0.30363
1	1.16715	-2.31854	-0.85585
8	1.28822	-1.73248	1.12432
1	1.03126	-2.62915	1.38389

Electric moments

0	0	1.46659994	0.537199974	2.46210003
0	1	-4.30999994E-02	0.136399999	8.47999975E-02
0	2	-3.22000012E-02	0.246600002	3.46999988E-02
0	3	8.32000002E-02	3.70999984E-02	-2.74999999E-02
0	4	3.44000012E-02	-8.79999995E-02	0.169000000
0	5	-0.241999999	-2.47000009E-02	-3.00999992E-02

0 6 0.182600006 -9.04999971E-02 -1.81000009E-02
0 7 6.65000007E-02 -0.210600004 -0.233799994
0 8 -0.141000003 0.186399996 -0.171800002
0 9 0.212899998 4.08000015E-02 0.248199999
0 10 -5.99999987E-02 -0.253199995 1.39999995E-03
0 11 -0.1897 -0.1787 -0.0653 0.0722 0.0139
0 12 0.2578 -0.1708 -0.0815 0.1023 0.0200
0 13 -0.0510 -0.0043 -0.0855 0.0099 0.0020
0 14 -0.0770 -0.1413 0.0987 0.0356 0.0071
0 15 0.0474 0.0267 -0.1323 0.0205 0.0041
0 16 0.0079 -0.1371 0.0601 0.0225 0.0045
1 1 -0.375699997 -5.38600016 3.92000005E-02
2 2 -0.502099991 -2.02649999 6.52839994
3 3 -1.94700003 -1.22230005 -2.05290008
4 4 1.36689997 4.30429983 1.06289995
5 5 1.01979995 -2.89949989 2.20499992
6 6 0.763400018 0.490599990 0.718500018
7 7 1.80149996 0.451099992 2.06019998
8 8 1.80920005 -3.37960005 0.798500001
9 9 1.02419996 -1.51329994 2.34010005
10 10 -2.24379992 -1.40540004 -0.960900009
11 11 -1.8194 0.4361 0.0553 Tot= 1.8717
12 12 0.6744 6.5079 1.0896 Tot= 6.6329
13 13 -0.2704 -2.9645 0.6525 Tot= 3.0475
14 14 -0.9991 3.2824 -0.4308 Tot= 3.4580
15 15 -3.3212 3.9599 -0.7607 Tot= 5.2240
16 16 1.1629 -0.8826 2.7225 Tot= 3.0892
Magnetic moments
1 -0.143000007 -0.356500000 -0.216000006
2 -0.568000019 -0.171900004 0.414999992
3 5.53000011E-02 -0.133000001 -0.631099999
4 -1.15000000E-02 5.07000014E-02 -0.520900011
5 0.263999999 0.117399998 -0.595600009
6 4.17999998E-02 -0.145699993 0.645500004
7 -0.404399991 -0.108099997 -0.273600012
8 0.219799995 0.257400006 -0.228200004
9 -0.395999998 -0.166700006 0.152899995
10 0.186199993 0.250299990 0.451099992
11 0.3550 0.1020 -0.2018
12 0.0672 -0.4116 -0.1535
13 -0.4389 -0.0909 0.1115
14 -0.2133 -0.2452 0.3722
15 -0.3994 0.0340 0.2179
16 -0.2167 -0.0535 0.2699
Energy ground
-686.913330
Excitation energies
6.57690001
6.94899988
6.96210003
7.19589996
7.27479982

7.56930017
7.63219976
7.68889999
7.71780014
7.78030014
7.8342
7.9866
8.0835
8.1410
8.2154
8.2411

Structure M-alpha
Number of atoms: 24

1	-3.78739	0.62761	-0.25619
8	-3.42335	-0.06962	0.30358
6	-2.4935	-0.84955	-0.4408
1	-2.71488	-1.90665	-0.26824
1	-2.57928	-0.65653	-1.51645
6	-1.07516	-0.56622	0.03245
1	-1.04411	-0.74819	1.11739
6	-0.58221	0.85716	-0.23946
1	-0.6319	1.05715	-1.31709
8	-1.37465	1.86004	0.38556
1	-1.86648	1.46679	1.12336
6	0.8756	1.00686	0.1932
1	0.94047	0.86075	1.28271
8	1.37641	2.28025	-0.16103
1	0.89876	2.94141	0.36271
6	1.71522	-0.05177	-0.51035
1	1.64491	0.11408	-1.59103
8	3.08501	-0.02247	-0.18597
1	3.2123	0.42939	0.65951
6	1.12607	-1.43605	-0.21761
8	-0.22681	-1.50027	-0.6246
1	1.63988	-2.19598	-0.81144
8	1.26639	-1.6869	1.15655
1	1.26768	-2.64048	1.31747

Electric moments

0	0	-1.88409996	-1.37179995	0.977800012		
0	1	0.178599998	0.104300000	0.121699996		
0	2	0.192499995	2.74999999E-02	-1.86999999E-02		
0	3	-7.58000016E-02	3.70000005E-02	-2.29000002E-02		
0	4	-1.78999994E-02	-0.175500005	0.261500001		
0	5	-0.142000005	4.50999998E-02	-0.242699996		
0	6	6.44999966E-02	0.209099993	9.80000012E-03		
0	7	0.207300007	7.90999979E-02	-7.94999972E-02		
0	8	-0.107600003	-0.252499998	-0.215900004		
0	9	-0.171499997	0.241500005	0.108999997		
0	10	0.143399999	4.85000014E-02	0.234099999		
0	11	-0.0112	-0.0027	0.0347	0.0013	0.0003
0	12	0.2618	0.1733	-0.1454	0.1197	0.0236

0 13 0.0906 -0.1296 0.0838 0.0320 0.0064
0 14 0.1718 -0.0074 -0.1253 0.0453 0.0091
0 15 0.0816 0.1056 0.2082 0.0611 0.0124
0 16 -0.0183 -0.1730 -0.1965 0.0689 0.0140
1 1 1.67700005 -4.14160013 -0.181199998
2 2 -0.837700009 -4.27409983 2.52999999E-02
3 3 -4.08230019 4.32550001 -1.88240004
4 4 -3.27390003 -1.15470004 -1.24000001
5 5 -1.36059999 -2.76830006 0.543399990
6 6 4.17999998E-02 -1.06019998 0.648100019
7 7 -3.30590010 4.52159977 -0.452399999
8 8 2.56809998 -1.77419996 0.388200015
9 9 3.15000005E-02 3.06719995 1.67530000
10 10 -0.123000003 0.911099970 1.91989994
11 11 -2.3999 5.2599 0.3867 Tot= 5.7944
12 12 -2.7788 -2.3033 -0.1884 Tot= 3.6142
13 13 -1.9654 -1.5954 0.3540 Tot= 2.5561
14 14 -1.8195 0.1083 0.5064 Tot= 1.8917
15 15 -0.9968 1.5128 1.6602 Tot= 2.4573
16 16 -1.1351 0.1047 0.5572 Tot= 1.2688
Magnetic moments
1 0.119199999 -0.602100015 0.522300005
2 -0.104699999 9.66000035E-02 0.611699998
3 -0.161500007 0.115300000 0.649699986
4 3.13000008E-02 0.110799998 3.39000002E-02
5 0.415399998 2.29000002E-02 -0.475499988
6 -0.279100001 -0.189700007 -0.101499997
7 5.86000010E-02 -7.85000026E-02 -0.293399990
8 0.278400004 0.248300001 -1.59999996E-03
9 1.99999995E-04 0.284599990 0.119800001
10 9.22999978E-02 -0.391600013 -0.277700007
11 0.7761 0.0803 0.2098
12 -0.1558 -0.2922 0.4017
13 -0.4319 -0.0342 -0.0033
14 0.1279 0.4046 -0.2445
15 -0.0087 -0.1954 0.3618
16 0.2406 -0.3447 -0.2354
Ground energy
-686.917175
Excitation energies
6.61560011
6.92729998
7.07070017
7.23689985
7.40959978
7.54110003
7.56780005
7.66060019
7.76859999
7.82390022
8.0036
8.0351

8.1169
8.1836
8.2559
8.2704

Structure A-beta
Number of atoms: 24

1	4.1559	-1.15107	0.02443
8	3.43629	-0.76438	-0.48976
6	2.63646	0.06426	0.34187
1	2.99379	1.09931	0.30314
1	2.6476	-0.28075	1.38328
6	1.2114	0.0204	-0.16474
1	1.24486	0.03605	-1.26443
6	0.36906	1.2003	0.32366
1	0.33857	1.19404	1.42415
8	0.93582	2.41011	-0.1374
1	0.2235	3.06722	-0.1886
6	-1.04045	1.02356	-0.21186
1	-0.99565	1.08096	-1.31055
8	-1.83071	2.09364	0.28074
1	-2.67971	2.07639	-0.18619
6	-1.61693	-0.331	0.16721
1	-1.74014	-0.36721	1.25858
8	-2.87292	-0.45875	-0.48562
1	-3.55331	-0.72562	0.14433
6	-0.66686	-1.45841	-0.24887
8	0.61649	-1.19264	0.28552
1	-0.62601	-1.53753	-1.34241
8	-1.07453	-2.70204	0.21254
1	-1.04889	-2.69391	1.18257

Electric moments

0	0	-1.96259999	0.797900021	2.72530007		
0	1	-3.19999992E-03	-9.40999985E-02	0.116599999		
0	2	-0.140900001	-0.104400001	1.03000002E-02		
0	3	0.119199999	2.84000002E-02	-0.309199989		
0	4	-8.52999985E-02	0.128199995	4.28000018E-02		
0	5	0.100199997	0.195099995	0.115900002		
0	6	0.200100005	-7.02000037E-02	0.330700010		
0	7	3.46000008E-02	-6.04999997E-02	3.07000000E-02		
0	8	-9.61999968E-02	8.07000026E-02	0.174199998		
0	9	-0.128199995	0.168500006	6.31999969E-02		
0	10	5.88000007E-02	-3.48000005E-02	-0.159600005		
0	11	0.1064	0.0764	-0.0901	0.0253	0.0050
0	12	0.0683	0.0855	-0.3522	0.1360	0.0269
0	13	0.1399	0.0213	-0.1651	0.0473	0.0094
0	14	-0.0114	0.0194	0.0931	0.0092	0.0018
0	15	-0.0436	-0.2018	-0.0641	0.0467	0.0094
0	16	-0.0571	0.0945	-0.0326	0.0133	0.0027
1	1	4.94110012	2.17589998	-1.62759995		
2	2	-8.05329990	2.89919996	-0.316700011		
3	3	1.39900005	-1.39129996	2.36800003		

4	4	0.393599987	-2.13459992	1.25100005
5	5	2.14520001	-2.25219989	3.15129995
6	6	0.819400012	2.25670004	0.671500027
7	7	0.102200001	3.68390012	1.84700000
8	8	3.35990000	1.28489995	0.156100005
9	9	-4.45330000	2.00329995	-0.953499973
10	10	-2.45560002	-1.80890000	1.06770003

11	11	2.7616	2.2590	-0.6619	Tot=	3.6287
12	12	1.9541	1.7881	0.7825	Tot=	2.7619
13	13	5.0497	1.5275	0.8854	Tot=	5.3494
14	14	-1.1996	0.3961	0.9234	Tot=	1.5649
15	15	2.3083	-3.3504	0.4567	Tot=	4.0941
16	16	2.4514	0.5345	1.6985	Tot=	3.0298

Magnetic moments

1	0.467700005	0.187500000	-0.140300006
2	0.279799998	4.99999989E-03	0.648199975
3	6.44999966E-02	-2.40000002E-02	-0.169499993
4	0.792699993	-5.09999990E-02	0.522099972
5	5.05000018E-02	0.379099995	0.736899972
6	-0.258700013	-9.09999982E-02	-0.493900001
7	6.97000027E-02	0.101700000	-0.365900010
8	-0.151199996	-8.33000019E-02	0.246999994
9	-0.131400004	-0.187999994	-1.72000006E-02
10	0.323500007	-0.110399999	-2.55999994E-02
11	0.2092	-0.1475	-0.2744
12	0.2254	-0.0518	0.2642
13	0.1634	0.0792	0.1799
14	-0.2771	0.0252	0.2505
15	0.2088	0.0507	0.7211
16	-0.1264	0.0309	0.4103

Ground energy
-686.916870
Excitation energies
6.52860022
6.69379997
6.99090004
7.35790014
7.42530012
7.55189991
7.66109991
7.79750013
7.91200018
7.94110012
8.0263
8.0770
8.0962
8.1075
8.2388
8.3137

Structure B-beta
Number of atoms: 24

1	-2.88814	-1.61132	0.44563
8	-3.45228	-0.91317	0.08193
6	-2.63094	0.15194	-0.36038
1	-3.09578	1.09294	-0.05812
1	-2.55851	0.12561	-1.45702
6	-1.23303	0.08129	0.22055
1	-1.30672	0.08627	1.32147
6	-0.35558	1.26686	-0.19431
1	-0.3779	1.36353	-1.28852
8	-0.89128	2.42557	0.41989
1	-0.77147	3.18554	-0.16438
6	1.08804	1.04491	0.2341
1	1.13691	1.08797	1.33283
8	1.88485	2.07499	-0.31741
1	2.80743	1.77665	-0.2877
6	1.54989	-0.32711	-0.23154
1	1.51349	-0.3758	-1.32672
8	2.88167	-0.53894	0.2287
1	3.42225	-0.88853	-0.49145
6	0.61095	-1.40552	0.29905
8	-0.67651	-1.16179	-0.20009
1	0.60162	-1.38945	1.40283
8	0.96654	-2.67211	-0.16977
1	1.63684	-3.04401	0.42025

Electric moments

0	0	4.30749989	-6.01999983E-02	-0.347299993		
0	1	5.05999997E-02	-5.88999987E-02	4.14000005E-02		
0	2	0.185200006	2.30999999E-02	4.78000008E-02		
0	3	-9.08999965E-02	-1.92000009E-02	-6.21999986E-02		
0	4	-2.29000002E-02	-1.84000004E-02	-0.251599997		
0	5	1.75999999E-02	0.222200006	0.345200002		
0	6	-0.161899999	-0.220100001	0.185800001		
0	7	9.96999964E-02	1.04999999E-02	-9.18999985E-02		
0	8	2.41000000E-02	0.106500000	-0.249200001		
0	9	0.185800001	-7.53000006E-02	2.00999994E-02		
0	10	-0.105800003	1.42000001E-02	-4.14999984E-02		
0	11	-0.0262	-0.0323	0.3309	0.1112	0.0218
0	12	-0.0180	-0.0341	0.0252	0.0021	0.0004
0	13	-0.0304	0.2526	-0.2522	0.1283	0.0255
0	14	-0.0133	-0.0298	-0.0840	0.0081	0.0016
0	15	-0.1960	-0.0366	-0.1408	0.0596	0.0119
0	16	0.0758	0.1291	-0.0734	0.0278	0.0056
1	1	-2.88689995	2.22650003	1.78999996		
2	2	3.22239995	2.73110008	-2.44140005		
3	3	4.30310011	-2.84089994	1.06309998		
4	4	3.59999990	2.25169992	-2.41720009		
5	5	-0.894999981	8.96999985E-02	0.493499994		
6	6	2.12680006	1.50940001	-1.47730005		
7	7	3.48180008	1.61020005	1.21280003		
8	8	2.69799995	-5.76090002	4.43639994		
9	9	-1.14639997	0.532999992	2.73620009		
10	10	2.15000010	-1.83570004	1.61280000		

11 11	1.8868	1.6274	-3.1638	Tot=	4.0272
12 12	-14.7072	2.5612	1.0698	Tot=	14.9668
13 13	0.2300	0.6880	-2.1686	Tot=	2.2867
14 14	-0.3708	-2.1189	-1.8544	Tot=	2.8401
15 15	0.9531	1.5450	0.7251	Tot=	1.9547
16 16	-2.1025	0.3644	1.0337	Tot=	2.3710

Magnetic moments

1	-0.435200006	0.217500001	0.158199996
2	-0.195700005	-3.09999995E-02	-0.145699993
3	-0.155100003	0.590799987	-0.777100027
4	-0.484600008	0.518299997	8.30999985E-02
5	-0.298400015	0.198300004	-0.172999993
6	0.423099995	0.250699997	0.551800013
7	0.373899996	-0.234599993	0.251300007
8	0.242200002	0.191900000	9.21000019E-02
9	-0.454800010	0.248400003	-0.115500003
10	8.07000026E-02	-0.178299993	-0.469099998
11	-0.1118	0.0806	0.6719
12	0.0144	-0.0487	-0.0079
13	0.0890	-0.0291	-0.1948
14	-0.1567	0.0815	-0.1092
15	0.2654	-0.0942	0.2881
16	-0.2906	-0.3396	-0.6574

Ground energy

-686.914551

Excitation energies

6.53100014
6.87729979
7.02899981
7.09740019
7.25600004
7.52710009
7.66669989
7.68160009
7.77610016
7.85680008
7.9844
8.0571
8.1145
8.1306
8.1624
8.2327

Structure C-beta

Numer of atoms: 24

1	4.1763	-1.03844	0.22508
8	3.41699	-0.823	-0.33073
6	2.63894	0.18581	0.29118
1	3.02377	1.18448	0.0544
1	2.62561	0.05829	1.38182
6	1.21895	0.07847	-0.21991
1	1.24139	0.07074	-1.32061

6	0.33639	1.22618	0.26987
1	0.34438	1.2304	1.37058
8	0.83526	2.45216	-0.22671
1	0.19169	3.13927	0.00773
6	-1.08569	0.98821	-0.21139
1	-1.09157	1.04762	-1.30786
8	-1.91198	2.02261	0.32437
1	-2.53548	2.31921	-0.35155
6	-1.59823	-0.38631	0.19873
1	-1.65457	-0.43291	1.29656
8	-2.86592	-0.636	-0.37963
1	-3.55456	-0.28151	0.19837
6	-0.60853	-1.45996	-0.26645
8	0.66723	-1.13914	0.2622
1	-0.56458	-1.49646	-1.36269
8	-0.94442	-2.73583	0.14476
1	-1.19076	-2.72147	1.08183

Electric moments

0	0	-1.81369996	2.95009995	2.43729997		
0	1	-9.65000018E-02	2.85999998E-02	-5.49999997E-03		
0	2	-0.130199999	-8.87999982E-02	4.14000005E-02		
0	3	2.41999999E-02	0.145999998	-0.211199999		
0	4	0.151999995	-3.46000008E-02	-7.78999999E-02		
0	5	0.162900001	0.177399993	0.227300003		
0	6	0.149700001	-7.66000003E-02	-4.08000015E-02		
0	7	0.204099998	-4.80000004E-02	0.275999993		
0	8	-5.29000014E-02	-0.183100000	-4.00000019E-03		
0	9	5.11999987E-02	8.11000019E-02	-0.183100000		
0	10	3.04000005E-02	8.38999972E-02	-5.99000007E-02		
0	11	-0.0320	-0.0373	0.0559	0.0055	0.0011
0	12	-0.0834	0.1452	-0.1490	0.0503	0.0099
0	13	0.0460	0.1224	-0.0205	0.0175	0.0035
0	14	-0.1127	-0.0106	0.1306	0.0299	0.0059
0	15	0.1379	0.1649	-0.3577	0.1742	0.0351
0	16	-0.0644	-0.0214	-0.1976	0.0436	0.0089
1	1	4.32100010	-0.469799995	2.46309996		
2	2	-8.05459976	4.06780005	-0.714699984		
3	3	2.42219996	2.31419992	5.27999997E-02		
4	4	0.925599992	-0.711099982	0.890200019		
5	5	1.51810002	1.81540000	0.209700003		
6	6	7.01070023	-2.99040008	2.39199996		
7	7	1.44579995	3.83769989	-1.96019995		
8	8	0.424100012	2.23930001	1.97189999		
9	9	-1.13820004	3.82540011	4.45009995		
10	10	-7.89669991	7.94999972E-02	-1.82009995		
11	11	2.4198	0.4318	-0.2619	Tot=	2.4720
12	12	4.8117	1.9321	1.1962	Tot=	5.3213
13	13	9.1492	-5.8787	3.9386	Tot=	11.5664
14	14	-3.1334	1.2481	0.8569	Tot=	3.4800
15	15	1.4394	0.4709	2.6302	Tot=	3.0350
16	16	-6.4290	4.7403	1.2308	Tot=	8.0819

Magnetic moments

1	-0.424299985	0.242599994	0.104300000
2	0.322299987	0.167799994	0.613399982
3	-3.33000012E-02	-0.129099995	0.235799998
4	-0.555800021	-2.96000000E-02	2.08999999E-02
5	0.171200007	0.402500004	0.863499999
6	-2.08000001E-02	-1.31000001E-02	0.161100000
7	-3.42000015E-02	-0.204099998	-0.443599999
8	0.259900004	-0.225400001	-0.701399982
9	0.263900012	0.152600005	6.44999966E-02
10	2.45999992E-02	-8.34999979E-02	-0.159899995
11	-0.2729	-0.4646	-0.5456
12	-0.0703	0.1457	0.4022
13	-0.1349	0.0352	-0.3939
14	0.2468	-0.2602	-0.3225
15	0.3238	-0.1936	-0.0832
16	0.1785	0.4825	0.1209

Ground energy
-686.914185
Excitation energy
6.53910017
6.63290024
6.90450001
7.17360020
7.35270023
7.44619989
7.58419991
7.60260010
7.74289989
7.86730003
7.9736
8.0091
8.0862
8.1158
8.2232
8.2976

Structure D-beta
Number of atoms : 24

1	-3.33438	-1.00951	1.09992
8	-3.4963	-0.89882	0.15264
6	-2.63072	0.08983	-0.37359
1	-3.02905	1.09529	-0.19068
1	-2.59949	-0.07883	-1.45333
6	-1.21489	0.02445	0.17532
1	-1.2625	0.00151	1.28443
6	-0.38365	1.24938	-0.23864
1	-0.43964	1.37547	-1.32466
8	-0.90929	2.44625	0.30443
1	-1.03143	2.34355	1.26068
6	1.08233	1.06299	0.14927
1	1.16343	1.19619	1.23982
8	1.85109	2.04126	-0.52012

1	2.57219	2.33345	0.05202
6	1.61677	-0.32877	-0.17204
1	1.7389	-0.39868	-1.2636
8	2.86786	-0.43297	0.4881
1	3.44484	-1.05184	0.02166
6	0.65997	-1.43103	0.27638
8	-0.61672	-1.17192	-0.28196
1	0.59483	-1.47176	1.37168
8	1.05922	-2.69401	-0.13563
1	1.35643	-2.65429	-1.05778

Electric moments

0	0	2.57489991	0.460000008	1.63269997		
0	1	-0.162900001	2.22999994E-02	0.136199996		
0	2	8.38000029E-02	8.13999996E-02	0.119900003		
0	3	1.48999998E-02	0.150800005	-8.48999992E-02		
0	4	0.153600007	-0.104000002	-0.235400006		
0	5	-4.16000001E-02	4.58000004E-02	-0.283300012		
0	6	-4.34000008E-02	-0.135700002	7.60000013E-03		
0	7	0.163299993	5.27000017E-02	0.300799996		
0	8	-9.95000005E-02	-8.00999999E-02	-5.93999997E-02		
0	9	-0.231800005	-0.100599997	3.83000001E-02		
0	10	0.208800003	-9.45999995E-02	-7.50999972E-02		
0	11	0.0365	0.2131	0.0185	0.0471	0.0090
0	12	0.0078	-0.0934	0.1102	0.0209	0.0041
0	13	0.0078	0.1085	0.0233	0.0124	0.0025
0	14	0.1059	0.0458	-0.0900	0.0214	0.0043
0	15	-0.0988	-0.0420	0.0145	0.0117	0.0023
0	16	0.0188	-0.0354	0.2657	0.0722	0.0145
1	1	2.06890011	0.218400002	-2.51519990		
2	2	-1.85230005	3.39809990	3.44160008		
3	3	2.87949991	7.40000010E-02	-3.05240011		
4	4	0.134499997	-3.40109992	-2.90450001		
5	5	3.74900007	0.637700021	-2.58929992		
6	6	0.130199999	1.99290001	2.16599989		
7	7	-2.75889993	4.99639988	5.38049984		
8	8	-1.33819997	1.22950006	4.01809978		
9	9	-0.543500006	0.576300025	1.00769997		
10	10	-0.773299992	0.201199993	1.97479999		
11	11	-1.2443	1.5187	1.1798	Tot=	2.2906
12	12	3.6391	-1.8365	-0.0313	Tot=	4.0764
13	13	1.4537	-1.9835	1.1390	Tot=	2.7102
14	14	-0.0184	-0.3761	1.0750	Tot=	1.1391
15	15	0.0510	-2.2825	-0.8569	Tot=	2.4385
16	16	0.4197	-0.2732	0.4721	Tot=	0.6882

Magnetic moments

1	0.289400011	0.247299999	8.29999987E-03
2	0.127399996	-0.338600010	-0.245199993
3	0.303900003	-0.273699999	0.583899975
4	0.321099997	-3.86999995E-02	0.145099998
5	5.99000007E-02	-0.108199999	-0.656700015
6	0.494700015	-9.29000005E-02	0.797599971
7	0.290899992	-6.92000017E-02	0.311600000

8	-0.154300004	-2.30000005E-03	0.478500009
9	0.290800005	0.271699995	0.136000007
10	0.210999995	9.97999981E-02	-0.576399982
11	0.0298	0.1955	0.0399
12	0.2863	0.4854	-0.2370
13	0.2312	-0.5288	0.1965
14	0.0157	0.1236	0.2496
15	-0.1261	-0.2080	-0.2459
16	0.2951	-0.3088	-0.0602

Ground energy
-686.907166
Excitation energies
6.58300018
6.65199995
6.81669998
6.98579979
7.22030020
7.39890003
7.47700024
7.60249996
7.77330017
7.80410004
7.8409
7.9048
8.0827
8.1024
8.1537
8.2044

Structure E-beta
Number of atoms: 24

1	-3.62984	-0.18827	0.98552
8	-3.57219	-0.33685	0.03128
6	-2.42786	-1.09932	-0.27505
1	-2.37134	-1.13648	-1.36605
1	-2.51971	-2.13171	0.09039
6	-1.11482	-0.53357	0.26712
1	-1.14262	-0.50287	1.36968
6	-0.72969	0.84905	-0.25371
1	-0.79193	0.8368	-1.35332
8	-1.61148	1.81477	0.27923
1	-1.2135	2.6856	0.11763
6	0.69892	1.19094	0.17132
1	0.70573	1.30162	1.26319
8	1.03126	2.47737	-0.33804
1	1.62503	2.40097	-1.09765
6	1.70013	0.10376	-0.18876
1	1.80761	0.02528	-1.28016
8	2.94585	0.37041	0.43066
1	3.63331	0.51636	-0.22966
6	1.18036	-1.24516	0.29645
8	-0.13447	-1.47161	-0.17302

1 1.18044 -1.26834 1.39936
8 1.99594 -2.23344 -0.23225
1 1.63519 -3.0985 0.01399
Electric moments
0 0 2.22210002 -0.983099997 -0.283399999
0 1 4.96999994E-02 -0.157299995 -0.104699999
0 2 9.70000029E-02 2.64999997E-02 9.60000046E-03
0 3 -9.65000018E-02 2.62000002E-02 -0.113600001
0 4 7.15999976E-02 -5.88999987E-02 -0.157299995
0 5 -6.36999980E-02 -0.118000001 -0.204400003
0 6 0.183300003 0.128900006 -0.221100003
0 7 5.88999987E-02 -3.68999988E-02 0.493000001
0 8 2.86999997E-02 -0.272700012 0.242500007
0 9 -9.10999998E-02 -7.84000009E-02 -8.56000036E-02
0 10 -5.29000014E-02 9.84999985E-02 -1.86000001E-02
0 11 -0.0376 0.2330 -0.0494 0.0581 0.0112
0 12 0.1409 0.0706 0.0829 0.0317 0.0062
0 13 -0.1977 -0.0753 0.1618 0.0709 0.0139
0 14 0.0665 -0.1550 0.1212 0.0431 0.0086
0 15 0.0958 -0.0810 -0.0860 0.0231 0.0046
0 16 0.0443 0.1313 -0.0513 0.0218 0.0044
1 1 -3.58150005 -3.86910009 4.42329979
2 2 3.29600000 -1.12769997 -3.05870008
3 3 -2.22320008 -2.52789998 2.13280010
4 4 1.49170005 6.15430021 -3.73289990
5 5 1.50650001 -4.38819981 0.573300004
6 6 -0.926599979 0.360300004 -1.35790002
7 7 0.660700023 0.956700027 -0.978200018
8 8 -1.66380000 0.797900021 0.872399986
9 9 -1.39880002 -0.150000006 2.74810004
10 10 0.766399980 0.307000011 0.636500001
11 11 2.1289 -1.7725 0.1382 Tot= 2.7736
12 12 -6.4505 -2.3404 1.8775 Tot= 7.1141
13 13 -1.3081 -0.5543 0.9327 Tot= 1.6995
14 14 1.6247 2.7046 2.3539 Tot= 3.9364
15 15 1.6909 -2.2586 -1.4355 Tot= 3.1656
16 16 0.4065 -0.1278 0.1414 Tot= 0.4490
Magnetic moments
1 -0.175899997 -0.158999994 0.372000009
2 0.396400005 -0.237200007 0.349299997
3 7.66000003E-02 -0.208000004 -0.469099998
4 0.563799977 -7.00000003E-02 -0.264099985
5 0.720600009 0.164399996 -0.148499995
6 5.29000014E-02 0.417299986 0.403100014
7 0.220599994 0.183899999 0.478599995
8 0.262899995 6.27999976E-02 -5.77000007E-02
9 6.00000005E-03 -0.388399988 0.247600004
10 3.18000019E-02 -1.68999992E-02 -0.769599974
11 0.0474 0.1469 -0.0158
12 -0.4949 0.0617 0.1281
13 -0.3848 -0.1636 -0.1010
14 -0.1383 0.1944 -0.1352

15	-0.2460	-0.0568	0.1370
16	0.1262	-0.0778	-0.1211

Ground energy
-686.908813
Excitation energy
6.55070019
6.72760010
6.76469994
7.06669998
7.29640007
7.53039980
7.56400013
7.67180014
7.79330015
7.82000017
7.8548
7.9584
8.0275
8.1047
8.1324
8.1751

Structure F-beta
Number of atoms: 24

1	-4.25725	-0.57146	0.11458
8	-3.37425	-0.38107	0.45782
6	-2.39813	-1.15295	-0.24027
1	-2.52686	-1.06328	-1.32583
1	-2.45506	-2.2103	0.04051
6	-1.04434	-0.60653	0.17564
1	-1.03608	-0.59077	1.28086
6	-0.72461	0.79884	-0.34437
1	-0.64736	0.74393	-1.4381
8	-1.70733	1.77613	-0.04801
1	-2.36154	1.40669	0.56682
6	0.6341	1.24402	0.19215
1	0.56054	1.34619	1.28946
8	1.02864	2.46576	-0.38876
1	0.48267	3.17086	-0.01103
6	1.67982	0.18958	-0.15314
1	1.8437	0.19845	-1.23412
8	2.95381	0.44257	0.4181
1	2.87022	0.5067	1.38221
6	1.21626	-1.21927	0.23476
8	-0.05532	-1.50408	-0.28996
1	1.17512	-1.30776	1.33966
8	2.05741	-2.18903	-0.30384
1	2.94962	-2.03348	0.04349

Electric moments

0	0	-2.83209991	-0.828800023	3.02839994
0	1	6.53000027E-02	-5.18000014E-02	0.226199999
0	2	3.62000018E-02	7.15999976E-02	9.73000005E-02

0 3 7.77999982E-02 8.47999975E-02 -9.43000019E-02
0 4 -7.06000030E-02 0.101499997 0.100900002
0 5 -7.76000023E-02 -8.20000023E-02 0.212699994
0 6 -5.22000007E-02 4.61999997E-02 0.157900006
0 7 7.98000023E-02 -7.31000006E-02 0.176400006
0 8 -8.91000032E-02 -0.165199995 -0.335999995
0 9 -0.210099995 9.51000005E-02 0.261299998
0 10 -1.72000006E-02 -2.70000007E-03 0.102600001
0 11 0.0805 0.1977 -0.1610 0.0715 0.0139
0 12 -0.0569 -0.2952 0.0548 0.0934 0.0183
0 13 0.2430 0.0847 0.2983 0.1552 0.0306
0 14 0.1391 0.0406 0.2838 0.1015 0.0203
0 15 -0.1087 -0.1274 -0.0159 0.0283 0.0057
0 16 -0.0627 0.1005 -0.1409 0.0339 0.0069
1 1 -6.70389986 -0.749499977 -2.75390005
2 2 0.589600027 -2.29229999 0.894599974
3 3 -0.680199981 -0.574699998 0.992299974
4 4 -2.34870005 0.238000005 0.178900003
5 5 -5.18629980 -0.233500004 0.524900019
6 6 4.60000010E-03 0.799799979 9.70000029E-03
7 7 5.00920010 -1.03120005 2.44600010
8 8 3.02519989 3.72000001E-02 0.739000022
9 9 -4.97770023 2.09969997 0.112999998
10 10 -1.05060005 2.08240008 3.02270007
11 11 -1.0384 -0.5755 2.8007 Tot= 3.0419
12 12 -1.6833 1.0547 2.5453 Tot= 3.2287
13 13 -3.6109 -1.0654 -0.8406 Tot= 3.8575
14 14 -1.1535 -2.2737 2.0241 Tot= 3.2553
15 15 -1.6123 -0.3142 3.4917 Tot= 3.8588
16 16 2.2958 -1.9154 2.0072 Tot= 3.6011
Magnetic moments
1 -0.273400009 4.38000001E-02 0.209600002
2 8.08999985E-02 -0.225600004 0.507300019
3 0.216499999 0.550499976 0.409999996
4 4.16000001E-02 -0.107500002 -0.388999999
5 0.392300010 0.214499995 0.252000004
6 0.218500003 0.438699991 -0.217399999
7 -0.323799998 -0.134000003 0.290499985
8 0.656199992 0.150500000 -3.06000002E-02
9 0.443399996 0.290300012 -0.363499999
10 0.462599993 -0.308800012 -0.613799989
11 0.0343 -0.3151 0.3152
12 0.0657 -0.0001 0.1363
13 -0.3586 -0.4668 0.1282
14 0.2426 0.4268 0.5301
15 0.2522 -0.1302 -0.0760
16 0.1107 -0.1954 0.0936
Ground energy
-686.911133
Excitation energy
6.55649996
6.68820000

6.87039995
7.11369991
7.24590015
7.32929993
7.40040016
7.50339985
7.69670010
7.74779987
7.9574
7.9941
8.0378
8.1635
8.1969
8.2712

Structure G-beta
Numer of atoms 24

1	-3.27138	0.5061	0.01696
8	-3.50779	-0.42642	0.14681
6	-2.38733	-1.24123	-0.12019
1	-2.32691	-1.48393	-1.19163
1	-2.52316	-2.17842	0.42449
6	-1.04761	-0.62643	0.28906
1	-1.01128	-0.54837	1.38859
6	-0.7643	0.76007	-0.29893
1	-0.76499	0.6899	-1.39565
8	-1.76465	1.67622	0.12255
1	-1.46364	2.57432	-0.08289
6	0.618	1.21294	0.17002
1	0.58386	1.28404	1.27019
8	0.9498	2.48426	-0.37241
1	0.76987	3.1843	0.26833
6	1.66735	0.18464	-0.22398
1	1.76593	0.15149	-1.31235
8	2.96009	0.48612	0.27085
1	2.89942	0.80353	1.18424
6	1.24626	-1.21298	0.25316
8	-0.05199	-1.52325	-0.17188
1	1.29418	-1.25257	1.35907
8	2.0587	-2.19414	-0.30984
1	2.90059	-2.21471	0.16711

Electric moments

0	0	2.23090005	3.47189999	3.14569998
0	1	3.59999994E-03	5.79000004E-02	0.272799999
0	2	-0.162900001	0.104699999	-6.37999997E-02
0	3	-9.79000032E-02	3.04000005E-02	-0.144099995
0	4	-4.19000015E-02	8.10000021E-03	2.43999995E-02
0	5	0.140499994	0.102600001	0.223900005
0	6	-0.175300002	-0.142000005	0.203700006
0	7	-0.125400007	0.146599993	-0.139500007
0	8	-3.04000005E-02	0.199699998	0.245800003
0	9	-0.160099998	-6.71999976E-02	-0.300700009

0 10 -0.141000003 8.91000032E-02 1.35000004E-02
0 11 0.0480 0.0196 -0.1619 0.0289 0.0056
0 12 0.0818 -0.1468 0.1146 0.0414 0.0080
0 13 0.2644 0.0087 0.0638 0.0740 0.0146
0 14 0.0586 -0.2050 -0.2839 0.1261 0.0250
0 15 -0.0203 0.0935 0.1320 0.0266 0.0053
0 16 -0.0171 -0.1395 0.3208 0.1227 0.0247
1 1 -0.282599986 -0.489399999 -2.06900001
2 2 1.16869998 -2.26690006 -0.117399998
3 3 0.183400005 -3.72259998 2.15350008
4 4 0.798900008 -1.02330005 1.12730002
5 5 -0.426200002 5.73999994E-02 0.125300005
6 6 1.28320003 1.38290000 1.20599997
7 7 0.442699999 -2.00550008 0.496300012
8 8 -3.20140004 -2.35619998 -4.34000008E-02
9 9 -0.762099981 3.08629990 -0.887099981
10 10 -1.73969996 2.44840002 -1.32369995
11 11 1.8441 -1.9728 0.6675 Tot= 2.7817
12 12 -0.5171 2.8591 0.2999 Tot= 2.9209
13 13 0.2707 -0.9815 0.0516 Tot= 1.0194
14 14 -2.0504 2.3165 3.9850 Tot= 5.0448
15 15 2.3182 -0.4826 2.4949 Tot= 3.4397
16 16 -1.2268 1.7333 3.8022 Tot= 4.3550
Magnetic moments
1 -0.239999995 -2.84000002E-02 1.75999999E-02
2 8.35999995E-02 -0.442299992 -0.240899995
3 0.220500007 0.400200009 -6.43000007E-02
4 9.48999971E-02 -0.256099999 0.151600003
5 -0.192100003 9.60000008E-02 6.34000003E-02
6 0.409099996 0.490000010 0.131400004
7 0.159299999 -4.65000011E-02 -1.01300001
8 0.297600001 8.20999965E-02 -0.263999999
9 0.398799986 0.555499971 -0.109999999
10 0.232199997 6.76999986E-02 -0.136299998
11 -0.7003 -0.0343 0.4523
12 -0.3574 -0.0573 0.2048
13 -0.1022 -0.2423 0.4111
14 -0.2178 0.5478 -0.0389
15 0.0563 0.0856 0.0379
16 0.2458 -0.0046 0.3389
Ground energy
-686.908386
Excitation energy
6.45230007
6.57000017
6.89010000
7.00339985
7.27069998
7.29969978
7.40570021
7.43889999
7.60580015

7.63950014
7.8845
7.9111
8.0361
8.0898
8.1802
8.2244

Structure H-beta
Number of atoms: 24

1	-4.31513	-0.73695	0.16055
8	-3.3699	-0.9098	0.26262
6	-2.63702	0.19478	-0.24409
1	-3.03995	1.14968	0.10984
1	-2.64696	0.19081	-1.34322
6	-1.20644	0.07975	0.23284
1	-1.19991	0.05153	1.33476
6	-0.33368	1.23583	-0.27317
1	-0.36905	1.23369	-1.36993
8	-0.84465	2.49115	0.13576
1	-0.38361	2.79451	0.93117
6	1.11186	1.02884	0.18953
1	1.13539	1.12096	1.28554
8	1.96206	2.05764	-0.27128
1	2.28975	1.84631	-1.15753
6	1.62113	-0.36973	-0.15494
1	1.75608	-0.44732	-1.24415
8	2.84148	-0.58684	0.52483
1	3.46846	-1.03585	-0.05574
6	0.60328	-1.43833	0.23412
8	-0.67166	-1.13163	-0.2823
1	0.54444	-1.51355	1.3332
8	1.01945	-2.63918	-0.32939
1	0.31147	-3.29139	-0.21215

Electric dipoles

0	0	-0.935400009	-0.327199996	-0.812399983		
0	1	-3.37999985E-02	-3.31999995E-02	-0.116599999		
0	2	1.90999992E-02	7.85000026E-02	0.188500002		
0	3	0.157900006	-1.44999996E-02	-4.08000015E-02		
0	4	-4.19999985E-03	-0.116899997	0.267899990		
0	5	3.11999992E-02	-4.41000015E-02	-0.209299996		
0	6	-2.92000007E-02	0.123000003	-0.294900000		
0	7	-5.66999987E-02	8.81000012E-02	0.200700000		
0	8	-0.109700002	-0.127599999	0.210999995		
0	9	0.217899993	0.173600003	-5.05000018E-02		
0	10	5.57000004E-02	0.180800006	0.172399998		
0	11	0.0232	0.0453	-0.0514	0.0052	0.0010
0	12	-0.0729	0.1491	-0.3909	0.1804	0.0354
0	13	0.0509	-0.0685	0.0180	0.0076	0.0015
0	14	-0.1435	-0.0539	-0.0437	0.0254	0.0051
0	15	-0.1067	-0.0101	0.1717	0.0410	0.0083
0	16	0.1618	0.0770	-0.0721	0.0373	0.0075

1	1	5.90660000	-0.808399975	-0.250999987
2	2	-7.38019991	-0.498299986	3.73790002
3	3	-5.15990019	5.92000000E-02	3.53839993
4	4	-2.11330009	-4.89419985	-5.14540005
5	5	-0.175699994	5.86639977	-2.56940007
6	6	-0.763800025	-0.274500012	3.10260010
7	7	0.189500004	-4.32530022	-4.88880014
8	8	8.51109982	1.59619999	-1.29960001
9	9	-2.17960000	-1.76419997	-2.56340003
10	10	-0.853999972	-0.821799994	1.12170005

11 11 -1.4705 1.8414 -0.5525 Tot= 2.4204
12 12 -5.0484 -1.9405 -0.9566 Tot= 5.4925
13 13 -3.8534 0.4323 1.5641 Tot= 4.1811
14 14 -3.2916 -0.2605 0.0846 Tot= 3.3030
15 15 0.2330 -1.8398 0.8736 Tot= 2.0500
16 16 4.2883 -1.6473 -0.6043 Tot= 4.6334

Magnetic moments

1	-0.395399988	0.563199997	-0.358500004
2	0.333099991	-0.172900006	-0.289600015
3	5.68999983E-02	0.339199990	0.301099986
4	-0.301499993	0.202000007	-0.203999996
5	0.360100001	-0.345800012	-0.382699996
6	-0.567799985	-7.00000022E-03	-0.709500015
7	-0.263099998	-0.158099994	-0.109600000
8	0.165600002	0.207399994	-5.77999987E-02
9	0.269699991	0.290699989	0.120200001
10	-0.458299994	-0.268999994	-0.279300004
11	-0.3657	-0.1481	0.2937
12	-0.3195	0.4795	-0.0289
13	-0.2311	0.5159	0.1243
14	-0.0843	-0.1362	-0.1317
15	0.1462	0.1308	-0.3932
16	-0.0123	-0.4558	-0.0745

Ground energy

-686.909546

Excitation energies

6.65280008
6.72030020
6.95889997
7.01859999
7.07940006
7.39709997
7.59870005
7.63549995
7.81759977
7.89519978
7.9611
8.0167
8.0322
8.1402
8.2274
8.2504

Structure I-beta

Number of atoms: 24

1	2.01017	-1.15414	1.46561
8	2.85111	-0.87362	1.07386
6	2.63812	-0.61627	-0.30222
1	2.91896	-1.49924	-0.89125
1	3.27686	0.22157	-0.59155
6	1.19131	-0.27684	-0.62027
1	1.07729	-0.18984	-1.71134
6	0.70402	1.01351	0.03871
1	0.90799	0.95389	1.11883
8	1.42489	2.08475	-0.53285
1	1.08587	2.90733	-0.14598
6	-0.79604	1.21217	-0.1697
1	-0.98204	1.37893	-1.23722
8	-1.21301	2.41011	0.46846
1	-1.49419	2.222	1.37583
6	-1.553	-0.02584	0.27802
1	-1.43395	-0.16128	1.36423
8	-2.91999	0.10273	-0.06211
1	-3.455	-0.38224	0.58049
6	-0.9597	-1.26842	-0.39026
8	0.41532	-1.37134	-0.12793
1	-1.12991	-1.207	-1.47766
8	-1.55198	-2.41961	0.13606
1	-2.18105	-2.78393	-0.49965

Electric moments

	0	0 -3.5738		-0.6952		0.0590
0	1	0.1226	0.0697	-0.0088		
0	2	-0.0485	-0.0082	-0.1031		
0	3	0.1724	-0.0524	0.2543		
0	4	-0.0507	0.2185	0.0486		
0	5	0.2003	-0.1275	0.1610		
0	6	0.0724	-0.0261	-0.1806		
0	7	0.0457	0.2589	0.2701		
0	8	0.0399	-0.0124	0.0242		
0	9	-0.2442	0.2243	0.3434		
0	10	0.2232	-0.0379	0.0837		
0	11	-0.0123	-0.1977	0.3588	0.1679	0.0325
0	12	0.0418	-0.0001	0.1759	0.0327	0.0064
0	13	0.0184	0.0172	0.0128	0.0008	0.0002
0	14	0.0905	0.1954	0.0007	0.0464	0.0092
0	15	0.0554	0.0621	-0.0589	0.0104	0.0021
0	16	0.1845	-0.0335	0.0781	0.0413	0.0083

1	1	2.7954	1.6324	-2.5168
2	2	-0.8764	-2.6507	-5.0286
3	3	-0.8448	1.6352	1.5639
4	4	-0.9014	-0.7572	-4.2577
5	5	-2.5055	-3.9624	-2.0718
6	6	-1.1725	1.0540	3.0876
7	7	-1.1252	-0.2122	-0.3808

8 8 -2.4310 2.4185 0.3046
9 8 -2.3898 -0.9685 1.0763
10 10 3.6999 3.2659 -0.2157
11 11 -2.7610 -4.8578 -1.0707 Tot= 5.6893
12 12 6.8788 1.7031 0.9743 Tot= 7.1532
13 13 3.4922 -0.4857 2.2734 Tot= 4.1951
14 14 5.6361 1.5318 -0.9529 Tot= 5.9178
15 15 -2.3813 3.3151 -0.1082 Tot= 4.0831
16 16 0.7503 1.3856 -1.4207 Tot= 2.1216

Magnetic moments

1	-0.3942	-0.1726	0.1635
2	0.0162	0.3829	-0.2490
3	-0.1224	-0.1334	-0.2130
4	0.6016	0.2109	-0.3561
5	-0.5917	0.2084	0.4015
6	-0.2116	0.4537	-0.7566
7	0.3853	-0.3446	0.4637
8	0.4190	0.1405	-0.2443
9	0.0229	0.2812	0.0274
10	-0.2001	-0.1638	0.5161
11	-0.2847	0.1846	-0.3264
12	-0.1210	-0.0116	-0.3761
13	0.2078	0.2902	0.0690
14	-0.0435	0.0106	0.3901
15	0.1255	-0.4070	0.2181
16	-0.3292	0.4224	-0.2089

Ground energy
-686.913747333

Excitation energies
6.6989
6.8369
6.9332
7.1430
7.3650
7.5693
7.5975
7.6623
7.7318
7.8570
7.8984
7.9707
8.0070
8.0765
8.1480
8.2153

Structure L-beta
Numer of atoms: 24

1	3.75242	-0.48807	1.21293
8	2.85902	-0.80313	1.02767
6	2.59645	-0.68849	-0.36284
1	2.82076	-1.63788	-0.86498

1	3.19017	0.10964	-0.82082
6	1.13459	-0.35854	-0.58574
1	0.97759	-0.28848	-1.67582
6	0.72578	0.97147	0.04475
1	0.95845	0.92086	1.11796
8	1.46925	2.00689	-0.57064
1	1.06342	2.85067	-0.31596
6	-0.7678	1.19607	-0.14679
1	-0.95714	1.36584	-1.21293
8	-1.17305	2.41358	0.48358
1	-1.35301	2.24696	1.42197
6	-1.58086	-0.00624	0.30035
1	-1.52378	-0.13649	1.39173
8	-2.93034	0.07399	-0.13032
1	-3.35972	0.84307	0.27078
6	-1.01829	-1.27957	-0.33757
8	0.34464	-1.40901	-0.05446
1	-1.17667	-1.22781	-1.43007
8	-1.62191	-2.41698	0.19543
1	-2.51261	-2.48922	-0.17792

Electric moments

```
         0          0 -0.478700012       2.53850007       7.49999983E-03
 0          1   3.26999985E-02  -4.80999984E-02   7.24000037E-02
 0          2  0.108099997     -0.138200000      -1.33999996E-02
 0          3  -5.31999990E-02  0.101300001      0.241200000
 0          4 -0.121799998       8.91000032E-02 -0.130300000
 0          5   2.30999999E-02 -0.187500000      -2.89999996E-03
 0          6  0.327399999       7.63000026E-02  0.182300001
 0          7  -4.91999984E-02  0.127299994     -0.286799997
 0          8  0.132300004       6.62000030E-02 -0.146400005
 0          9   6.80000009E-03   5.40000014E-03  0.162200004
 0         10  0.142800003     -0.130500004     -0.143199995
 0     11        0.0374     -0.2320     -0.2075      0.0983      0.0191
 0     12        0.0972     -0.0849     -0.2422      0.0753      0.0148
 0     13       -0.1356      0.0292      0.2430      0.0783      0.0155
 0     14       -0.1240      0.1062     -0.1098      0.0387      0.0077
 0     15        0.0113     -0.2252      0.1234      0.0661      0.0132
 0     16        0.0077     -0.0171     -0.1286      0.0169      0.0034
         1          1   3.36490011     -0.585399985      -4.55670023
         2          2  -6.95060015       1.46190000      -1.39660001
         3          3   4.50129986      -3.47079992      -4.55929995
         4          4   4.45839977       6.25050020       3.69330001
         5          5   1.43760002      -1.40030003      -2.52340007
         6          6   1.80209994      -1.29260004      -2.85780001
         7          7  -2.69429994       1.53970003       3.78049994
         8          8   2.43470001       1.14269996      0.711000025
         9          9  -3.90899992      0.370900005      -2.76220012
        10         10   2.94589996      0.243599996       3.29999998E-02
11 11    -0.5324      -1.0242       -0.4369  Tot=             1.2342
12 12   -0.5092       1.6315       -0.9209  Tot=             1.9414
13 13   -0.6990       2.5132       -0.4400  Tot=             2.6455
14 14    1.5993       0.0043       -1.9312  Tot=             2.5074
```

15 15 0.4621 0.5944 -0.1513 Tot= 0.7679
16 16 4.4124 2.9995 0.2734 Tot= 5.3423
Magnetic moments
1 9.22999978E-02 -0.478700012 0.129600003
2 -0.472999990 -0.170300007 0.398299992
3 0.259000003 -5.79000004E-02 -4.58000004E-02
4 0.410299987 -6.92000017E-02 0.151400000
5 -0.440800011 2.05000006E-02 -0.256999999
6 -0.238800004 0.195099995 0.754400015
7 0.191599995 0.300099999 -0.117399998
8 0.239999995 -0.472999990 -0.117899999
9 0.482699990 -0.535600007 0.151700005
10 -0.382699996 -0.316100001 0.490500003
11 0.0967 -0.1434 -0.0999
12 -0.1254 -0.5080 0.5180
13 0.1144 -0.3297 -0.5135
14 0.3617 -0.0333 -0.0326
15 -0.2656 -0.3287 -0.1876
16 -0.1890 -0.2585 -0.0726
Ground energy
-686.912781
Excitation energies
6.63110018
6.75180006
6.95539999
7.04920006
7.17439985
7.32740021
7.59420013
7.63180017
7.74940014
7.82490015
7.9265
8.0130
8.0812
8.0979
8.1479
8.2420

Structure M-beta
Number of atoms: 24
8 0.33161 -1.41254 -0.1097
6 1.12673 -0.34809 -0.60831
6 0.71218 0.96036 0.0621
6 -0.77921 1.21414 -0.14358
6 -1.59869 0.00664 0.27244
6 -1.04708 -1.27256 -0.36794
6 2.58325 -0.69356 -0.3825
8 2.82613 -0.77259 1.01393
8 1.47718 2.01288 -0.51457
8 -1.20874 2.33916 0.60584
8 -2.93222 0.21035 -0.14361

8 -1.68964 -2.4065 0.12426
1 3.75811 -0.97747 1.16494
1 2.78924 -1.65585 -0.8684
1 3.19466 0.08768 -0.84871
1 0.97762 -0.25288 -1.69705
1 0.92262 0.87978 1.13409
1 1.56082 2.72833 0.12987
1 -0.9566 1.39242 -1.21449
1 -0.97192 3.1433 0.12423
1 -1.53807 -0.09579 1.36712
1 -3.50194 -0.42739 0.30913
1 -1.24527 -1.2569 -1.44811
1 -1.40693 -2.53292 1.04493
Electric moments
0 0 2.75679994 0.881299973 0.435200006
0 1 -6.36000037E-02 -0.163000003 -1.95000004E-02
0 2 -2.45999992E-02 -0.114200003 -1.30000003E-02
0 3 3.06000002E-02 9.83999968E-02 -0.170000002
0 4 0.257200003 -4.06000018E-02 0.145999998
0 5 1.16999997E-02 -3.70999984E-02 0.192399994
0 6 -0.104000002 4.28000018E-02 0.228100002
0 7 -0.224299997 0.202900007 0.105899997
0 8 -2.39000004E-02 0.102899998 0.358000010
0 9 3.70999984E-02 -6.41999990E-02 0.258300006
0 10 -0.267100006 1.74000002E-02 -0.316199988
0 11 0.1408 -0.3039 0.0930 0.1208 0.0236
0 12 -0.0290 0.1600 0.0182 0.0268 0.0053
0 13 0.1468 0.0508 -0.0638 0.0282 0.0056
0 14 -0.0356 -0.0242 0.0212 0.0023 0.0005
0 15 0.0133 0.1570 0.0250 0.0254 0.0051
1 1 1.16069996 -5.94280005 -0.169799998
2 2 -3.85190010 2.17659998 -0.718299985
3 3 7.56829977 3.88409996 -4.50089979
4 4 1.59360003 -3.36279988 1.14069998
5 5 -1.62520003 2.63969994 5.25309992
6 6 3.56570005 -1.26839995 -1.51820004
7 7 -3.96160007 -5.57350016 -2.20009995
8 8 2.53660011 1.99140000 -2.69449997
9 9 0.668200016 2.91930008 1.12849998
10 10 4.77010012 -0.631900012 -1.34230006
11 11 3.9230 -1.0552 1.7379 Tot= 4.4186
12 12 2.6306 -2.1540 1.8831 Tot= 3.8867
13 13 1.0141 -0.4850 0.7136 Tot= 1.3315
14 14 -3.2371 0.5905 -0.0715 Tot= 3.2912
15 15 3.4093 4.9077 -1.9359 Tot= 6.2815
Magnetic moments
1 0.126699999 0.330900013 1.60000008E-02
2 -0.535600007 0.127399996 0.423900008
3 -0.342299998 -0.153699994 0.127200007
4 -0.274399996 -0.225999996 0.702199996
5 -4.54999991E-02 -0.209000006 -0.485000014
6 0.380800009 -0.361900002 9.69000012E-02

7	0.334600002	-0.393999994	-0.132400006
8	0.157600001	-0.188999996	0.858600020
9	-0.429199994	-0.201499999	-1.20999999E-02
10	0.134900004	-0.220200002	0.246299997
11	-0.3924	-0.1690	0.5282
12	-0.1067	-0.0892	-0.3008
13	-0.0199	-0.0715	-0.3726
14	0.2486	0.0896	-0.2095
15	0.0059	0.0812	0.3421

Ground energies
-686.915222

Excitation energies
6.61899982
6.68550014
6.78919983
7.21280003
7.29180002
7.33750010
7.52720022
7.65910006
7.74329996
7.90719986
7.9729
8.0239
8.0831
8.1406
8.1532

D-galactose

Structure A-alpha
Number of atoms : 24

1	-4.25772	-0.13224	-0.35469
8	-3.33559	-0.16198	-0.64017
6	-2.57542	0.65188	0.23164
1	-2.64765	0.31059	1.27096
1	-2.8803	1.70658	0.18515
6	-1.12511	0.5377	-0.19862
1	-1.01515	0.97384	-1.19994
6	-0.65306	-0.91787	-0.27348
1	-1.16535	-1.40263	-1.10974
8	-0.98055	-1.62095	0.91041
1	-0.16008	-1.87924	1.35069
6	0.85509	-0.92533	-0.51906
1	1.03086	-0.55431	-1.54021
8	1.31869	-2.25478	-0.37578
1	2.26632	-2.23934	-0.57856
6	1.60975	-0.01706	0.45132
1	1.55148	-0.43296	1.46037
8	2.98505	0.0389	0.12673

1	3.0345	0.42651	-0.76203
6	0.99213	1.37663	0.47084
8	-0.37962	1.31599	0.73682
1	1.42107	1.97844	1.2788
8	1.28494	1.94791	-0.78574
1	1.05851	2.88837	-0.75466

Electric moments

0	0	0.274899989	3.36190009	-0.648899972
0	1	-0.107100002	2.38000005E-02	-2.48000007E-02
0	2	-7.02999979E-02	-4.50999998E-02	9.92999971E-02
0	3	-3.72000001E-02	1.72000006E-02	-0.123700000
0	4	5.90999983E-02	0.150700003	4.50999998E-02
0	5	7.94000030E-02	9.48000029E-02	0.149399996
0	6	-5.07999994E-02	-0.367000014	-0.284999996
0	7	0.158999994	-7.24000037E-02	-7.71000013E-02
0	8	0.121900000	6.52000010E-02	-3.75999995E-02
0	9	0.261799991	-7.69999996E-03	0.146500006
0	10	2.09999997E-02	2.59000007E-02	-0.226099998
0	11	-6.67999983E-02	-0.174099997	-6.31000027E-02
0	12	4.10000002E-03	6.44999966E-02	-0.138500005
0	13	1.73000004E-02	1.35000004E-02	1.53999999E-02
0	14	-6.09999988E-03	0.240700006	-0.234799996
0	15	1.62000004E-02	3.48999985E-02	0.127200007
0	16	0.136600003	0.255100012	-0.111100003
1	1	2.24659991	2.00920010	0.132799998
2	2	0.886600018	1.77610004	0.642899990
3	3	-2.68339992	-0.663900018	-4.17999998E-02
4	4	-1.41970003	4.45639992	-3.96359992
5	5	-0.969200015	2.46749997	0.359200001
6	6	-2.62639999	2.74099994	1.69620001
7	7	1.72230005	-2.94289994	0.225600004
8	8	5.90030003	1.40330005	-2.01889992
9	9	-2.46309996	-1.82729995	0.730700016
10	10	-1.28090000	0.166500002	-0.191499993
11	11	-5.09940004	-4.51580000	1.14779997
12	12	-1.78919995	0.227899998	-0.489100009
13	13	-1.53960001	5.99999987E-02	1.09440005
14	14	-1.62269998	1.21930003	-1.93840003
15	15	0.494300008	-1.80960000	-0.820900023
16	16	-4.29899979	3.69449997	2.30259991

Magnetic moments

1	-0.393999994	0.378800005	5.62000014E-02
2	0.103799999	-8.51999968E-02	-0.680400014
3	-0.326099992	-7.42999986E-02	0.172199994
4	0.238100007	-0.328000009	0.272799999
5	-0.268900007	-0.269100010	-0.176300004
6	-0.542400002	-0.466899991	0.491299987
7	-0.499199986	0.405800015	0.131200001
8	-0.493299991	7.00000022E-03	-0.137999997
9	0.539499998	8.20999965E-02	0.255400002
10	8.56999978E-02	-5.68999983E-02	-4.41999994E-02
11	-0.315299988	-0.143399999	-0.669900000

12	-0.241899997	-0.110600002	-0.174999997
13	-0.315200001	0.461400002	0.100199997
14	-8.88999999E-02	0.391900003	0.517400026
15	1.44999996E-02	-0.193000004	2.11999994E-02
16	-3.90000008E-02	0.163100004	0.235100001

Ground energy
-686.918701
Excitation energies
6.70289993
6.70919991
7.00150013
7.18069983
7.35290003
7.54949999
7.65329981
7.70310020
7.79820013
7.84240007
7.92749977
8.08520031
8.11789989
8.21560001
8.25189972
8.31130028

B.alpha
Number of atoms: 24

1	3.10312	0.94187	-0.72548
8	3.50647	0.09988	-0.47664
6	2.57731	-0.6352	0.29332
1	2.56787	-0.30091	1.33755
1	2.89671	-1.67912	0.26887
6	1.14668	-0.54956	-0.23074
1	1.09417	-0.97984	-1.2387
6	0.62091	0.89012	-0.31016
1	1.0846	1.37291	-1.18978
8	0.95184	1.61786	0.85319
1	0.16225	2.12054	1.10976
6	-0.89268	0.87636	-0.52097
1	-1.11387	0.49365	-1.52459
8	-1.45561	2.17543	-0.34507
1	-0.99042	2.79746	-0.92494
6	-1.57952	-0.02214	0.4882
1	-1.38785	0.36734	1.49768
8	-2.96888	-0.1106	0.25168
1	-3.24167	0.7162	-0.17327
6	-0.97696	-1.42076	0.40141
8	0.38823	-1.35099	0.66964
1	-1.40583	-2.06707	1.17492
8	-1.18085	-1.96622	-0.882
1	-2.07303	-2.33869	-0.90473

Electric moments

0	0	-2.98379993	2.15520000	-1.79310000
0	1	7.54999965E-02	0.141000003	6.57000020E-02
0	2	6.00000005E-03	5.79999993E-03	0.229599997
0	3	-0.100900002	0.177000001	-0.240300000
0	4	-1.99999995E-04	-3.17000002E-02	9.99999978E-03
0	5	-0.171299994	7.40000010E-02	0.255100012
0	6	0.170100003	0.247899994	-0.264899999
0	7	0.116999999	-5.31999990E-02	-0.144800007
0	8	4.03999984E-02	5.00000007E-02	2.12999992E-02
0	9	4.83000018E-02	-5.07999994E-02	0.125599995
0	10	7.64999986E-02	0.186600000	8.54000002E-02
0	11	6.70000017E-02	-2.67999992E-02	-1.32999998E-02
0	12	0.137500003	0.147599995	-0.148200005
0	13	-6.44000024E-02	-7.05000013E-02	-0.211099997
0	14	-2.25000009E-02	0.142299995	0.243799999
0	15	-0.126699999	-4.65999991E-02	0.197200000
0	16	-0.150000006	5.40000014E-02	-0.106700003
1	1	-2.06979990	-3.71620011	1.88479996
2	2	-1.96770000	-4.11040020	1.96389997
3	3	-1.81560004	-3.66849995	2.23320007
4	4	7.72999972E-02	2.31850004	-3.03000007E-02
5	5	0.123400003	1.70829999	0.182400003
6	6	-1.44480002	-1.27769995	-1.79310000
7	7	-2.20619988	-1.93219995	0.518899977
8	8	1.43519998	-3.08540010	1.93770003
9	9	0.749700010	0.377799988	-0.203400001
10	10	-1.12520003	-2.21679997	0.334800005
11	11	-0.226699993	1.11389995	-1.58260000
12	12	-2.16599989	-1.97479999	-1.12349999
13	13	-1.35730004	0.695699990	-2.92740011
14	14	-2.24040008	2.28360009	-0.388099998
15	15	-0.715799987	1.10270000	1.11510003
16	16	-1.23060000	1.07260001	1.57630002

Magnetic moments

1	-0.426699996	0.463400006	-0.171399996
2	0.218099996	0.407400012	-0.253600001
3	0.333400011	-7.97000006E-02	-0.405499995
4	0.559499979	-0.378399998	-0.150800005
5	-0.174199998	-0.256900012	0.239199996
6	0.177900001	-0.322800010	0.290199995
7	0.297300011	0.323700011	-0.304300010
8	-0.137600005	-0.143399999	-0.118500002
9	0.314500004	-0.584699988	-0.259499997
10	0.434700012	-0.513100028	-0.259600013
11	0.356599987	0.500500023	-0.221399993
12	-3.77000012E-02	-0.236200005	0.501399994
13	-0.488700002	-0.243200004	-0.219899997
14	-0.102799997	4.90000006E-03	0.288100004
15	-6.62000030E-02	0.151199996	-0.188199997
16	-0.222299993	-0.501100004	-0.168899998

Ground energy

-686.913208

Excitation energies
6.80079985
6.93510008
7.04160023
7.22940016
7.34159994
7.64709997
7.73699999
7.77750015
7.82180023
7.88539982
7.99529982
8.03499985
8.07310009
8.17980003
8.20139980
8.22140026

C-Alpha
Number of atoms: 24

1	3.48558	1.01057	-0.22845
8	3.40415	0.16606	-0.69267
6	2.60007	-0.68005	0.10423
1	2.72943	-0.48049	1.17394
1	2.91369	-1.71046	-0.08017
6	1.12725	-0.53299	-0.26094
1	0.94724	-0.94959	-1.25749
6	0.6483	0.91396	-0.21079
1	1.17004	1.52	-0.95404
8	0.93472	1.49353	1.0593
1	0.747	0.8047	1.7182
6	-0.84662	0.93816	-0.49412
1	-0.99164	0.57815	-1.5198
8	-1.40021	2.24002	-0.48614
1	-1.39955	2.54452	0.43547
6	-1.62315	0.01293	0.43857
1	-1.59856	0.42869	1.45801
8	-2.95737	-0.10995	-0.01221
1	-3.18199	0.74199	-0.42257
6	-0.9864	-1.38395	0.48229
8	0.39836	-1.27739	0.71907
1	-1.36218	-1.94979	1.34161
8	-1.25727	-2.08177	-0.69488
1	-1.95466	-1.59069	-1.15996

Electric moments

0	0	-0.982599974	1.56429994	1.48319995
0	1	0.152400002	-1.75999999E-02	4.12999988E-02
0	2	-0.192900002	1.68999992E-02	-4.67000008E-02
0	3	-0.165700004	-7.11999983E-02	-0.125200003
0	4	0.127000004	0.130199999	-1.15999999E-02
0	5	0.112099998	6.06999993E-02	-0.197200000
0	6	0.179600000	0.133900002	-0.126200005

0 7 -0.307700008 -2.62000002E-02 0.209199995
0 8 -7.85000026E-02 4.47999984E-02 5.27999997E-02
0 9 0.178900003 -7.33999982E-02 0.468600005
0 10 7.60000013E-03 -0.282200009 0.113899998
0 11 -3.20000015E-02 1.76999997E-02 0.137899995
0 12 0.202099994 -0.198300004 -0.193599999
0 13 5.02999984E-02 -0.112700000 8.79999995E-02
0 14 -0.172199994 0.245399997 0.119999997
0 15 9.20000002E-02 4.43000011E-02 9.30000022E-02
0 16 0.108700000 0.234300002 1.37000000E-02
1 1 0.269499987 -1.71010005 -2.22639990
2 2 -2.93219995 -2.17319989 -7.55999982E-02
3 3 1.31110001 0.214399993 -0.452699989
4 4 1.90149999 -0.328799993 1.67030001
5 5 4.30000015E-02 -3.83000001E-02 2.22889996
6 6 -7.90000036E-02 0.218099996 4.16069984
7 7 1.62769997 -0.370700002 -0.908800006
8 8 2.42379999 -1.12199998 0.640799999
9 9 2.56150007 7.18000010E-02 -2.69580007
10 10 -1.00230002 1.04380000 2.89310002
11 11 -2.56410003 0.605700016 2.35560012
12 12 -1.09000001E-02 1.14110005 -1.52590001
13 13 -3.55559993 3.37360001 0.305700004
14 14 -0.169200003 2.69000006 1.82040000
15 15 1.90100002 2.03609991 1.49790001
16 16 -0.499599993 -0.454200000 -1.13460004
Magnetic moments
1 -5.20999990E-02 -0.131600007 -7.46999979E-02
2 0.108700000 -0.359200001 -0.592199981
3 -0.243799999 0.607599974 -0.547399998
4 0.172700003 0.364600003 -0.356799990
5 -0.380199999 -0.424100012 0.126100004
6 -0.230700001 0.399699986 -2.83000004E-02
7 -6.53000027E-02 -0.289700001 -0.459899992
8 -0.502499998 0.206100002 -0.426699996
9 0.272199988 8.72000009E-02 0.249799997
10 -0.308800012 1.49999997E-02 7.90000036E-02
11 9.71999988E-02 6.03000000E-02 -9.04999971E-02
12 -1.75999999E-02 0.769400001 0.307000011
13 -0.140599996 0.100500003 9.32999998E-02
14 -6.44000024E-02 0.519800007 -0.181700006
15 -0.118199997 -0.120999999 -0.119300000
16 -0.261599988 -0.182999998 0.138999999
Ground energy
-686.919922
Excitation energy
6.91459990
7.11460018
7.26590014
7.51149988
7.63509989
7.66930008

7.83279991
7.97310019
8.06369972
8.13239956
8.16800022
8.24050045
8.38980007
8.42780018
8.47630024
8.49409962

D-Alpha
Number of atoms: 24

1	-3.19106	0.74285	1.12144
8	-3.52586	-0.07032	0.71872
6	-2.62636	-0.47724	-0.295
1	-2.73606	0.12784	-1.20233
1	-2.87691	-1.51242	-0.53499
6	-1.17718	-0.43658	0.16906
1	-1.10526	-0.90446	1.15688
6	-0.5657	0.96314	0.20106
1	-1.04387	1.56691	0.98045
8	-0.82129	1.66632	-1.00942
1	-0.22569	1.32943	-1.69387
6	0.92937	0.91315	0.52565
1	1.04376	0.50486	1.5335
8	1.48586	2.20355	0.58112
1	1.41502	2.59571	-0.30195
6	1.65534	-0.03331	-0.42919
1	1.68491	0.39027	-1.4459
8	2.97331	-0.22828	0.05646
1	3.34605	-1.01402	-0.37344
6	0.90904	-1.38132	-0.47031
8	-0.4391	-1.2189	-0.78131
1	1.30388	-2.01188	-1.28062
8	1.04834	-2.02802	0.76723
1	1.86807	-1.69694	1.1709

Electric moments

0	0	3.07380009	-0.365599990	-1.98080003
0	1	6.56000003E-02	5.95000014E-02	0.152300000
0	2	8.26999992E-02	-9.49999988E-02	0.103900000
0	3	-7.54000023E-02	7.09000006E-02	-0.138999999
0	4	-6.91000000E-02	-7.40000000E-03	-0.107900001
0	5	-0.183799997	3.90000008E-02	5.20000011E-02
0	6	0.213799998	-1.30000000E-03	-0.313499987
0	7	0.318100005	-7.20999986E-02	-0.295800000
0	8	-7.94999972E-02	0.402700007	-9.60000046E-03
0	9	1.60000008E-02	3.88999991E-02	-0.184900001
0	10	9.91000012E-02	-0.101400003	-1.46000003E-02
0	11	-9.13000032E-02	0.108400002	-1.43999998E-02
0	12	-4.01000008E-02	0.188899994	0.158800006
0	13	-1.55999996E-02	-0.178499997	9.25000012E-02

0 14 0.138099998 -0.176799998 0.194000006
0 15 3.70999984E-02 5.38000017E-02 0.111699998
0 16 -2.95000002E-02 -8.03000033E-02 5.31000011E-02
1 1 -0.839699984 -1.73329997 3.47390008
2 2 2.06080008 -2.94819999 -3.45070004
3 3 -1.25059998 1.20000001E-02 -0.376700014
4 4 0.994499981 -2.75950003 3.29380012
5 5 -1.85759997 3.20790005 -0.536599994
6 6 3.36260009 -1.13049996 -4.67549992
7 7 2.50020003 -1.21229994 -1.66779995
8 8 2.77080011 -0.255499989 -1.20630002
9 9 -3.10170007 -1.53830004 2.89750004
10 10 -5.64930010 3.62240005 0.856199980
11 11 1.93250000 -0.457800001 -1.54620004
12 12 8.69000033E-02 -2.28080010 1.67530000
13 13 2.61960006 -0.269600004 -1.80460000
14 14 -1.24030006 3.14280009 0.848399997
15 15 1.52470005 -0.583500028 -2.32850003
16 16 2.61649990 0.999800026 0.918399990
Magnetic moments
1 -0.144099995 9.52999964E-02 -0.171299994
2 -0.315600008 -0.273799986 -0.104000002
3 0.188199997 0.630599976 0.474299997
4 -0.324299991 -2.16000006E-02 -0.538500011
5 -6.23000003E-02 0.415399998 0.175699994
6 -8.39999989E-02 -0.127900004 -8.10000021E-03
7 -0.460200012 -0.107199997 0.550000012
8 -0.196700007 -8.89999978E-03 -5.10000018E-03
9 -1.62000004E-02 0.639599979 0.218400002
10 -1.32999998E-02 0.154300004 -0.199399993
11 -0.666999996 -0.354099989 -0.381599993
12 -9.62999985E-02 5.33000007E-02 7.16999993E-02
13 0.282700002 8.57999995E-02 -8.70999992E-02
14 0.153400004 1.39999995E-03 0.386500001
15 0.265899986 0.366400003 2.36000009E-02
16 3.35000008E-02 -0.371499985 0.160799995
Ground energy
-686.905090
Excitation energy
6.79430008
6.87050009
6.91039991
7.10680008
7.41249990
7.52199984
7.61590004
7.83500004
7.86089993
7.87069988
7.94339991
7.98769999
8.13319969

8.19349957
8.30249977
8.33290005

E-Alpha
Number of atoms: 24

1	3.29334	-0.30728	-1.45475
8	3.49309	0.08436	-0.59242
6	2.60553	-0.47467	0.35809
1	2.68158	0.13224	1.25996
1	2.87555	-1.51009	0.6072
6	1.16999	-0.47125	-0.14604
1	1.1353	-0.93223	-1.14378
6	0.57762	0.92999	-0.2505
1	1.10527	1.46024	-1.05294
8	0.76327	1.60734	0.98664
1	0.73423	2.5535	0.7908
6	-0.91896	0.88743	-0.5746
1	-1.07519	0.49156	-1.5813
8	-1.44868	2.20154	-0.56947
1	-1.72657	2.40182	0.33529
6	-1.61033	-0.02595	0.42378
1	-1.52245	0.4162	1.42657
8	-2.97058	-0.17514	0.04276
1	-3.42737	-0.65737	0.74778
6	-0.9218	-1.39673	0.43553
8	0.42425	-1.28259	0.76052
1	-1.36	-2.03855	1.21379
8	-1.03113	-1.99898	-0.83359
1	-1.96777	-2.19518	-0.98172

Electric moments

0	0	-2.61129999	-1.01030004	0.853799999
0	1	4.69999984E-02	0.109999999	-0.129700005
0	2	-9.73000005E-02	8.42000023E-02	5.68999983E-02
0	3	-8.70999992E-02	4.43000011E-02	-4.85999994E-02
0	4	0.116200000	-9.01999995E-02	-0.105200000
0	5	0.185399994	-0.100699998	0.140499994
0	6	-0.160300002	-6.23999983E-02	0.251100004
0	7	1.43999998E-02	-0.223299995	7.40000010E-02
0	8	0.386700004	0.150600001	-6.97000027E-02
0	9	-0.292499989	-0.273799986	0.245700002
0	10	0.123999998	-7.32000023E-02	-0.161899999
0	11	8.60000029E-02	9.60000008E-02	0.141000003
0	12	8.16000029E-02	-0.105099998	-9.67999995E-02
0	13	-5.55999987E-02	2.42999997E-02	0.184599996
0	14	0.139300004	-0.275799990	-7.47999996E-02
0	15	1.72000006E-02	-7.84000009E-02	0.124100000
0	16	-2.00999994E-02	0.209900007	4.19999985E-03
1	1	4.05700016	1.52980006	-2.90319991
2	2	-2.00990009	-5.27549982	-0.439399987
3	3	-2.96600008	-0.426200002	4.31689978

4	4	0.358200014	1.13220000	0.804400027
5	5	0.816999972	-1.84759998	-1.36039996
6	6	-1.46159995	1.17429996	1.01820004
7	7	-0.845399976	-3.11540008	-0.549600005
8	8	2.43400002	1.29709995	-2.47679996
9	9	-0.465700001	-2.70560002	-0.108900003
10	10	1.65090001	1.71120000	-1.71889997
11	11	0.883199990	2.98070002	0.459500015
12	12	-4.06739998	-1.79929996	2.95910001
13	13	1.18589997	-2.67750001	-2.50830007
14	14	-2.38470006	-0.635800004	2.41240001
15	15	-1.00549996	-0.523800015	1.53849995
16	16	2.75359988	0.766900003	-1.12189996

Magnetic moments

1	6.76999986E-02	-0.102600001	0.483900011
2	-0.329899997	4.98000011E-02	1.37000000E-02
3	-0.300300002	-0.455100000	-0.410299987
4	0.208000004	0.230299994	-0.331600010
5	0.375000000	-0.294400007	0.263399988
6	0.259200007	-0.294600010	0.564899981
7	-0.254500002	0.126499996	0.290199995
8	-8.57999995E-02	-0.173199996	0.597700000
9	-0.122800000	-0.222100005	-0.833100021
10	0.134499997	-0.136500001	2.81000007E-02
11	3.07999998E-02	-9.12000015E-02	0.112300001
12	0.335500002	-0.397899985	9.13000032E-02
13	-4.82000001E-02	0.150700003	6.71000034E-02
14	0.302500010	-0.286199987	0.314799994
15	-0.376100004	0.278400004	0.382299989
16	5.88999987E-02	9.31999981E-02	0.331600010

Ground energy
-686.910461
Excitation energy
6.46339989
6.76270008
6.82200003
7.19649982
7.31580019
7.46129990
7.56090021
7.58419991
7.63430023
7.82800007
7.86180019
7.94780016
8.00520039
8.16269970
8.20219994
8.25409985

F_alpha
Number of atoms: 24

1	2.73308	-0.99348	-0.69824
8	3.19801	-0.14313	-0.6429
6	2.64586	0.57901	0.42247
1	2.91955	1.62911	0.28591
1	3.06994	0.25313	1.38937
6	1.12555	0.51713	0.56284
1	0.85912	0.98403	1.52062
6	0.53436	-0.8928	0.5719
1	0.84983	-1.39506	1.49913
8	0.98915	-1.62918	-0.55235
1	0.48295	-2.45824	-0.55349
6	-0.9918	-0.85369	0.54563
1	-1.35465	-0.44413	1.49955
8	-1.39343	-2.20977	0.39258
1	-2.35734	-2.26249	0.44375
6	-1.49214	0.01864	-0.59469
1	-1.19953	-0.43447	-1.54421
8	-2.90074	0.16537	-0.59911
1	-3.23021	0.03077	0.30003
6	-0.82561	1.39227	-0.48699
8	0.57214	1.28937	-0.50403
1	-1.08149	2.00454	-1.35764
8	-1.30056	1.98073	0.70137
1	-0.98515	2.89587	0.72389

Electric moments

0	0	-2.99710011	-0.151899993	3.61299992
0	1	0.149700001	1.72000006E-02	0.100400001
0	2	-8.60999972E-02	9.14999992E-02	0.194199994
0	3	3.42999995E-02	0.132900000	9.49999969E-03
0	4	-7.97000006E-02	0.209199995	-4.10000011E-02
0	5	0.149599999	-8.63000005E-02	-0.158800006
0	6	-0.122000001	-4.96000014E-02	-2.67999992E-02
0	7	0.167999998	-0.223000005	0.363599986
0	8	-1.89999994E-02	-8.10000002E-02	4.30999994E-02
0	9	-1.37000000E-02	-0.101300001	0.268999994
0	10	8.03000033E-02	-6.15000017E-02	0.197799996
0	11	-0.186000004	5.27999997E-02	-0.222200006
0	12	3.40999998E-02	0.134399995	-9.22999978E-02
0	13	6.06999993E-02	0.102100000	0.205599993
0	14	-6.98999986E-02	-9.30000003E-03	3.81000005E-02
0	15	0.199300006	-7.93000013E-02	0.118400000
0	16	-1.57999992E-02	3.22999991E-02	0.103200004
1	1	2.83620000	3.49880004	-0.186800003
2	2	-1.33879995	1.03199995	-0.656199992
3	3	1.33539999	1.86409998	0.664200008
4	4	-2.27690005	-5.80249977	1.58800006
5	5	-0.432999998	3.73469996	1.51390004
6	6	-1.10450006	-1.14800000	1.39859998
7	7	0.227799997	-0.647499979	1.07889998
8	8	7.61759996	1.56750000	0.830699980
9	9	1.71039999	-1.92600000	1.01010001
10	10	0.319700003	-1.46089995	2.62680006

11	11	-0.707300007	-1.97700000	1.38600004
12	12	-0.907800019	-0.231900007	0.819899976
13	13	0.229599997	1.14489996	0.294200003
14	14	-0.287800014	1.06710005	1.28020000
15	15	0.169100001	-3.74489999	0.961600006
16	16	3.23920012	3.66969991	0.808300018

Magnetic moments

1	0.180099994	-9.60000046E-03	0.567799985
2	-0.223499998	-9.35999975E-02	-8.47999975E-02
3	-0.497200012	0.146699995	0.461199999
4	7.41999969E-02	-2.71000005E-02	-0.174400002
5	0.370999992	-5.35999984E-02	-0.132400006
6	0.149399996	-0.454499990	-0.320699990
7	-9.20000020E-03	0.363599986	-0.255400002
8	-4.80000023E-03	0.225299999	-5.90000004E-02
9	0.627300024	0.676599979	-0.195999995
10	-0.377700001	0.503799975	-0.231199995
11	6.93000033E-02	0.302399993	-7.51999989E-02
12	0.247999996	0.515600026	6.84999973E-02
13	0.515200019	-0.541400015	0.251100004
14	-0.281100005	-0.454600006	0.411300004
15	9.64000002E-02	-0.244599998	0.139100000
16	0.229599997	3.22000012E-02	-3.24000008E-02

Ground energy
-686.918457
Excitation energy
6.51959991
6.91169977
6.99760008
7.09110022
7.45270014
7.49139977
7.56790018
7.61129999
7.70340014
7.82730007
7.87190008
7.89639997
7.97279978
8.03190041
8.12500000
8.16079998

G-Alpha
Number of atoms: 24

1	3.11323	-1.33575	-0.36872
8	3.58096	-0.49696	-0.2636
6	2.62442	0.50219	0.03635
1	2.87388	1.39391	-0.54893
1	2.6697	0.77624	1.09648
6	1.20953	0.07238	-0.29615
1	1.2191	-0.42431	-1.27729

6	0.21834	1.22718	-0.36447
1	0.47765	1.87457	-1.20655
8	0.31812	2.06228	0.78315
1	-0.01892	1.5729	1.54791
6	-1.20824	0.70851	-0.57355
1	-1.2428	0.21016	-1.54851
8	-2.13382	1.76221	-0.6584
1	-2.18134	2.18668	0.21105
6	-1.5565	-0.33503	0.49362
1	-1.57854	0.13676	1.4843
8	-2.82613	-0.909	0.33012
1	-2.84041	-1.31665	-0.5496
6	-0.47207	-1.41954	0.51939
8	0.81426	-0.88138	0.70092
1	-0.62628	-2.08859	1.37207
8	-0.59651	-2.11611	-0.69653
1	-0.01333	-2.88841	-0.66868

Electric moments

0	0	-0.263000011	-2.50720000	0.198400006
0	1	-0.115300000	-7.15999976E-02	-0.204500005
0	2	-3.40000005E-03	-7.45000020E-02	-6.58999979E-02
0	3	8.32000002E-02	-4.03999984E-02	-3.88999991E-02
0	4	-6.03000000E-02	4.98999991E-02	-4.69999984E-02
0	5	0.177599996	-7.81999975E-02	-3.55000012E-02
0	6	0.178299993	0.258399993	-0.293399990
0	7	1.97000001E-02	0.186600000	-0.103699997
0	8	-0.163599998	-4.45999987E-02	0.132499993
0	9	-0.214100003	-5.73999994E-02	-3.90999988E-02
0	10	2.96999998E-02	0.224399999	-0.150900006
0	11	-7.45000020E-02	-0.175500005	0.158099994
0	12	3.80000006E-03	-3.33999991E-02	0.275099993
0	13	-4.74999994E-02	0.267100006	-9.70000029E-03
0	14	-2.63999999E-02	0.125400007	-0.112700000
0	15	5.95999993E-02	0.176200002	1.41000003E-02
0	16	-0.117600001	7.19999988E-03	-0.125200003
1	1	3.92000005E-02	3.43039989	0.790400028
2	2	0.738799989	-3.41370010	-4.29320002
3	3	-0.134800002	1.39279997	-0.983799994
4	4	-2.86999997E-02	-2.65580010	-1.99199998
5	5	0.650500000	0.297899991	2.71259999
6	6	-1.33130002	1.15709996	-0.206900001
7	7	0.677500010	-1.15579998	2.64770007
8	8	-0.705299973	0.806999981	0.753700018
9	9	-2.65660000	2.63190007	1.12860000
10	10	-0.330599993	-3.76270008	-0.754400015
11	11	-1.36469996	-0.123800002	-1.03670001
12	12	3.08890009	-1.12609994	-1.46519995
13	13	-9.79000032E-02	0.393599987	0.482400000
14	14	4.46560001	-0.947899997	-0.217700005
15	15	1.36210001	-1.15590000	1.75610006
16	16	-0.883700013	-3.29520011	-1.06340003

Magnetic moments

1	-0.374799997	-0.358399987	0.147400007
2	-0.201000005	-0.298799992	0.174400002
3	-0.137999997	-0.619199991	-0.233400002
4	-0.154899999	-0.279799998	-0.432099998
5	0.166299999	-2.98999995E-02	0.477899998
6	0.201900005	-8.34999979E-02	0.524100006
7	-0.130199999	0.320100009	0.649699986
8	0.254099995	6.44999966E-02	-0.142800003
9	0.555700004	-1.67999994E-02	0.342700005
10	-2.52999999E-02	0.319299996	-6.49000034E-02
11	-0.693400025	-0.322299987	0.378399998
12	0.314799994	0.208600000	-8.89999978E-03
13	-0.173199996	6.47000000E-02	-0.214000002
14	0.153500006	-0.626999974	3.35000008E-02
15	-0.143500000	0.259099990	-0.188899994
16	0.231800005	2.05000006E-02	0.156200007

Ground energy
-686.914124
Excitation energy
6.73050022
6.85540009
6.93400002
7.13159990
7.35790014
7.53109980
7.53520012
7.70030022
7.77610016
7.85370016
7.96210003
8.04539967
8.08139992
8.17899990
8.19610023
8.25689983

A-Beta
Number of atoms: 24

1	-4.25772	-0.13224	-0.35469
8	-3.33559	-0.16198	-0.64017
6	-2.57542	0.65188	0.23164
1	-2.64765	0.31059	1.27096
1	-2.8803	1.70658	0.18515
6	-1.12511	0.5377	-0.19862
1	-1.01515	0.97384	-1.19994
6	-0.65306	-0.91787	-0.27348
1	-1.16535	-1.40263	-1.10974
8	-0.98055	-1.62095	0.91041
1	-0.16008	-1.87924	1.35069
6	0.85509	-0.92533	-0.51906

1	1.03086	-0.55431	-1.54021
8	1.31869	-2.25478	-0.37578
1	2.26632	-2.23934	-0.57856
6	1.60975	-0.01706	0.45132
1	1.55148	-0.43296	1.46037
8	2.98505	0.0389	0.12673
1	3.0345	0.42651	-0.76203
6	0.99213	1.37663	0.47084
8	-0.37962	1.31599	0.73682
1	1.42107	1.97844	1.2788
8	1.28494	1.94791	-0.78574
1	1.05851	2.88837	-0.75466

Electric moments

0	0	0.274899989	3.36190009	-0.648899972
0	1	-0.107100002	2.38000005E-02	-2.48000007E-02
0	2	-7.02999979E-02	-4.50999998E-02	9.92999971E-02
0	3	-3.72000001E-02	1.72000006E-02	-0.123700000
0	4	5.90999983E-02	0.150700003	4.50999998E-02
0	5	7.94000030E-02	9.48000029E-02	0.149399996
0	6	-5.07999994E-02	-0.367000014	-0.284999996
0	7	0.158999994	-7.24000037E-02	-7.71000013E-02
0	8	0.121900000	6.52000010E-02	-3.75999995E-02
0	9	0.261799991	-7.69999996E-03	0.146500006
0	10	2.09999997E-02	2.59000007E-02	-0.226099998
0	11	-6.67999983E-02	-0.174099997	-6.31000027E-02
0	12	4.10000002E-03	6.44999966E-02	-0.138500005
0	13	1.73000004E-02	1.35000004E-02	1.53999999E-02
0	14	-6.09999988E-03	0.240700006	-0.234799996
0	15	1.62000004E-02	3.48999985E-02	0.127200007
0	16	0.136600003	0.255100012	-0.111100003
1	1	2.24659991	2.00920010	0.132799998
2	2	0.886600018	1.77610004	0.642899990
3	3	-2.68339992	-0.663900018	-4.17999998E-02
4	4	-1.41970003	4.45639992	-3.96359992
5	5	-0.969200015	2.46749997	0.359200001
6	6	-2.62639999	2.74099994	1.69620001
7	7	1.72230005	-2.94289994	0.225600004
8	8	5.90030003	1.40330005	-2.01889992
9	9	-2.46309996	-1.82729995	0.730700016
10	10	-1.28090000	0.166500002	-0.191499993
11	11	-5.09940004	-4.51580000	1.14779997
12	12	-1.78919995	0.227899998	-0.489100009
13	13	-1.53960001	5.99999987E-02	1.09440005
14	14	-1.62269998	1.21930003	-1.93840003
15	15	0.494300008	-1.80960000	-0.820900023
16	16	-4.29899979	3.69449997	2.30259991

Magnetic moments

1	-0.393999994	0.378800005	5.62000014E-02
2	0.103799999	-8.51999968E-02	-0.680400014
3	-0.326099992	-7.42999986E-02	0.172199994
4	0.238100007	-0.328000009	0.272799999
5	-0.268900007	-0.269100010	-0.176300004

6	-0.542400002	-0.466899991	0.491299987
7	-0.499199986	0.405800015	0.131200001
8	-0.493299991	7.00000022E-03	-0.137999997
9	0.539499998	8.20999965E-02	0.255400002
10	8.56999978E-02	-5.68999983E-02	-4.41999994E-02
11	-0.315299988	-0.143399999	-0.669900000
12	-0.241899997	-0.110600002	-0.174999997
13	-0.315200001	0.461400002	0.100199997
14	-8.88999999E-02	0.391900003	0.517400026
15	1.44999996E-02	-0.193000004	2.11999994E-02
16	-3.90000008E-02	0.163100004	0.235100001

Ground energy
-686.918701
Exccitation energy
6.70289993
6.70919991
7.00150013
7.18069983
7.35290003
7.54949999
7.65329981
7.70310020
7.79820013
7.84240007
7.92749977
8.08520031
8.11789989
8.21560001
8.25189972
8.31130028

B-Beta
Number of atoms: 24

1	3.10312	0.94187	-0.72548
8	3.50647	0.09988	-0.47664
6	2.57731	-0.6352	0.29332
1	2.56787	-0.30091	1.33755
1	2.89671	-1.67912	0.26887
6	1.14668	-0.54956	-0.23074
1	1.09417	-0.97984	-1.2387
6	0.62091	0.89012	-0.31016
1	1.0846	1.37291	-1.18978
8	0.95184	1.61786	0.85319
1	0.16225	2.12054	1.10976
6	-0.89268	0.87636	-0.52097
1	-1.11387	0.49365	-1.52459
8	-1.45561	2.17543	-0.34507
1	-0.99042	2.79746	-0.92494
6	-1.57952	-0.02214	0.4882
1	-1.38785	0.36734	1.49768
8	-2.96888	-0.1106	0.25168
1	-3.24167	0.7162	-0.17327

6	-0.97696	-1.42076	0.40141
8	0.38823	-1.35099	0.66964
1	-1.40583	-2.06707	1.17492
8	-1.18085	-1.96622	-0.882
1	-2.07303	-2.33869	-0.90473

Electric moments

0	0	-2.98379993	2.15520000	-1.79310000
0	1	7.54999965E-02	0.141000003	6.57000020E-02
0	2	6.00000005E-03	5.79999993E-03	0.229599997
0	3	-0.100900002	0.177000001	-0.240300000
0	4	-1.99999995E-04	-3.17000002E-02	9.99999978E-03
0	5	-0.171299994	7.40000010E-02	0.255100012
0	6	0.170100003	0.247899994	-0.264899999
0	7	0.116999999	-5.31999990E-02	-0.144800007
0	8	4.03999984E-02	5.00000007E-02	2.12999992E-02
0	9	4.83000018E-02	-5.07999994E-02	0.125599995
0	10	7.64999986E-02	0.186600000	8.54000002E-02
0	11	6.70000017E-02	-2.67999992E-02	-1.32999998E-02
0	12	0.137500003	0.147599995	-0.148200005
0	13	-6.44000024E-02	-7.05000013E-02	-0.211099997
0	14	-2.25000009E-02	0.142299995	0.243799999
0	15	-0.126699999	-4.65999991E-02	0.197200000
0	16	-0.150000006	5.40000014E-02	-0.106700003
1	1	-2.06979990	-3.71620011	1.88479996
2	2	-1.96770000	-4.11040020	1.96389997
3	3	-1.81560004	-3.66849995	2.23320007
4	4	7.72999972E-02	2.31850004	-3.03000007E-02
5	5	0.123400003	1.70829999	0.182400003
6	6	-1.44480002	-1.27769995	-1.79310000
7	7	-2.20619988	-1.93219995	0.518899977
8	8	1.43519998	-3.08540010	1.93770003
9	9	0.749700010	0.377799988	-0.203400001
10	10	-1.12520003	-2.21679997	0.334800005
11	11	-0.226699993	1.11389995	-1.58260000
12	12	-2.16599989	-1.97479999	-1.12349999
13	13	-1.35730004	0.695699990	-2.92740011
14	14	-2.24040008	2.28360009	-0.388099998
15	15	-0.715799987	1.10270000	1.11510003
16	16	-1.23060000	1.07260001	1.57630002

Magnetic moments

1	-0.426699996	0.463400006	-0.171399996
2	0.218099996	0.407400012	-0.253600001
3	0.333400011	-7.97000006E-02	-0.405499995
4	0.559499979	-0.378399998	-0.150800005
5	-0.174199998	-0.256900012	0.239199996
6	0.177900001	-0.322800010	0.290199995
7	0.297300011	0.323700011	-0.304300010
8	-0.137600005	-0.143399999	-0.118500002
9	0.314500004	-0.584699988	-0.259499997
10	0.434700012	-0.513100028	-0.259600013
11	0.356599987	0.500500023	-0.221399993
12	-3.77000012E-02	-0.236200005	0.501399994

13 -0.488700002 -0.243200004 -0.219899997
14 -0.102799997 4.90000006E-03 0.288100004
15 -6.62000030E-02 0.151199996 -0.188199997
16 -0.222299993 -0.501100004 -0.168899998

Ground energy
-686.913208

Excitation energy
6.80079985
6.93510008
7.04160023
7.22940016
7.34159994
7.64709997
7.73699999
7.77750015
7.82180023
7.88539982
7.99529982
8.03499985
8.07310009
8.17980003
8.20139980
8.22140026

C-Beta

Number of atoms: 24

1	3.48558	1.01057	-0.22845
8	3.40415	0.16606	-0.69267
6	2.60007	-0.68005	0.10423
1	2.72943	-0.48049	1.17394
1	2.91369	-1.71046	-0.08017
6	1.12725	-0.53299	-0.26094
1	0.94724	-0.94959	-1.25749
6	0.6483	0.91396	-0.21079
1	1.17004	1.52	-0.95404
8	0.93472	1.49353	1.0593
1	0.747	0.8047	1.7182
6	-0.84662	0.93816	-0.49412
1	-0.99164	0.57815	-1.5198
8	-1.40021	2.24002	-0.48614
1	-1.39955	2.54452	0.43547
6	-1.62315	0.01293	0.43857
1	-1.59856	0.42869	1.45801
8	-2.95737	-0.10995	-0.01221
1	-3.18199	0.74199	-0.42257
6	-0.9864	-1.38395	0.48229
8	0.39836	-1.27739	0.71907
1	-1.36218	-1.94979	1.34161
8	-1.25727	-2.08177	-0.69488
1	-1.95466	-1.59069	-1.15996

Electric moments
0 0 -0.982599974 1.56429994 1.48319995

0	1	0.152400002	-1.75999999E-02	4.12999988E-02
0	2	-0.192900002	1.68999992E-02	-4.67000008E-02
0	3	-0.165700004	-7.11999983E-02	-0.125200003
0	4	0.127000004	0.130199999	-1.15999999E-02
0	5	0.112099998	6.06999993E-02	-0.197200000
0	6	0.179600000	0.133900002	-0.126200005
0	7	-0.307700008	-2.62000002E-02	0.209199995
0	8	-7.85000026E-02	4.47999984E-02	5.27999997E-02
0	9	0.178900003	-7.33999982E-02	0.468600005
0	10	7.60000013E-03	-0.282200009	0.113899998
0	11	-3.20000015E-02	1.76999997E-02	0.137899995
0	12	0.202099994	-0.198300004	-0.193599999
0	13	5.02999984E-02	-0.112700000	8.79999995E-02
0	14	-0.172199994	0.245399997	0.119999997
0	15	9.20000002E-02	4.43000011E-02	9.30000022E-02
0	16	0.108700000	0.234300002	1.37000000E-02
1	1	0.269499987	-1.71010005	-2.22639990
2	2	-2.93219995	-2.17319989	-7.55999982E-02
3	3	1.31110001	0.214399993	-0.452699989
4	4	1.90149999	-0.328799993	1.67030001
5	5	4.30000015E-02	-3.83000001E-02	2.22889996
6	6	-7.90000036E-02	0.218099996	4.16069984
7	7	1.62769997	-0.370700002	-0.908800006
8	8	2.42379999	-1.12199998	0.640799999
9	9	2.56150007	7.18000010E-02	-2.69580007
10	10	-1.00230002	1.04380000	2.89310002
11	11	-2.56410003	0.605700016	2.35560012
12	12	-1.09000001E-02	1.14110005	-1.52590001
13	13	-3.55559993	3.37360001	0.305700004
14	14	-0.169200003	2.69000006	1.82040000
15	15	1.90100002	2.03609991	1.49790001
16	16	-0.499599993	-0.454200000	-1.13460004

Magnetic moments

1	-5.20999990E-02	-0.131600007	-7.46999979E-02
2	0.108700000	-0.359200001	-0.592199981
3	-0.243799999	0.607599974	-0.547399998
4	0.172700003	0.364600003	-0.356799990
5	-0.380199999	-0.424100012	0.126100004
6	-0.230700001	0.399699986	-2.83000004E-02
7	-6.53000027E-02	-0.289700001	-0.459899992
8	-0.502499998	0.206100002	-0.426699996
9	0.272199988	8.72000009E-02	0.249799997
10	-0.308800012	1.49999997E-02	7.90000036E-02
11	9.71999988E-02	6.03000000E-02	-9.04999971E-02
12	-1.75999999E-02	0.769400001	0.307000011
13	-0.140599996	0.100500003	9.32999998E-02
14	-6.44000024E-02	0.519800007	-0.181700006
15	-0.118199997	-0.120999999	-0.119300000
16	-0.261599988	-0.182999998	0.138999999

Ground energy

-686.919922

Excitation energy

6.91459990
7.11460018
7.26590014
7.51149988
7.63509989
7.66930008
7.83279991
7.97310019
8.06369972
8.13239956
8.16800022
8.24050045
8.38980007
8.42780018
8.47630024
8.49409962

D-Beta
Number of atoms: 24

1	-3.19106	0.74285	1.12144
8	-3.52586	-0.07032	0.71872
6	-2.62636	-0.47724	-0.295
1	-2.73606	0.12784	-1.20233
1	-2.87691	-1.51242	-0.53499
6	-1.17718	-0.43658	0.16906
1	-1.10526	-0.90446	1.15688
6	-0.5657	0.96314	0.20106
1	-1.04387	1.56691	0.98045
8	-0.82129	1.66632	-1.00942
1	-0.22569	1.32943	-1.69387
6	0.92937	0.91315	0.52565
1	1.04376	0.50486	1.5335
8	1.48586	2.20355	0.58112
1	1.41502	2.59571	-0.30195
6	1.65534	-0.03331	-0.42919
1	1.68491	0.39027	-1.4459
8	2.97331	-0.22828	0.05646
1	3.34605	-1.01402	-0.37344
6	0.90904	-1.38132	-0.47031
8	-0.4391	-1.2189	-0.78131
1	1.30388	-2.01188	-1.28062
8	1.04834	-2.02802	0.76723
1	1.86807	-1.69694	1.1709

Electric moments

0	0	3.07380009	-0.365599990	-1.98080003
0	1	6.56000003E-02	5.95000014E-02	0.152300000
0	2	8.26999992E-02	-9.49999988E-02	0.103900000
0	3	-7.54000023E-02	7.09000006E-02	-0.138999999
0	4	-6.91000000E-02	-7.40000000E-03	-0.107900001
0	5	-0.183799997	3.90000008E-02	5.20000011E-02
0	6	0.213799998	-1.30000000E-03	-0.313499987
0	7	0.318100005	-7.20999986E-02	-0.295800000

0	8	-7.94999972E-02	0.402700007	-9.60000046E-03
0	9	1.60000008E-02	3.88999991E-02	-0.184900001
0	10	9.91000012E-02	-0.101400003	-1.46000003E-02
0	11	-9.13000032E-02	0.108400002	-1.43999998E-02
0	12	-4.01000008E-02	0.188899994	0.158800006
0	13	-1.55999996E-02	-0.178499997	9.25000012E-02
0	14	0.138099998	-0.176799998	0.194000006
0	15	3.70999984E-02	5.38000017E-02	0.111699998
0	16	-2.95000002E-02	-8.03000033E-02	5.31000011E-02
1	1	-0.839699984	-1.73329997	3.47390008
2	2	2.06080008	-2.94819999	-3.45070004
3	3	-1.25059998	1.20000001E-02	-0.376700014
4	4	0.994499981	-2.75950003	3.29380012
5	5	-1.85759997	3.20790005	-0.536599994
6	6	3.36260009	-1.13049996	-4.67549992
7	7	2.50020003	-1.21229994	-1.66779995
8	8	2.77080011	-0.255499989	-1.20630002
9	9	-3.10170007	-1.53830004	2.89750004
10	10	-5.64930010	3.62240005	0.856199980
11	11	1.93250000	-0.457800001	-1.54620004
12	12	8.69000033E-02	-2.28080010	1.67530000
13	13	2.61960006	-0.269600004	-1.80460000
14	14	-1.24030006	3.14280009	0.848399997
15	15	1.52470005	-0.583500028	-2.32850003
16	16	2.61649990	0.999800026	0.918399990

Magnetic moments

1	-0.144099995	9.52999964E-02	-0.171299994
2	-0.315600008	-0.273799986	-0.104000002
3	0.188199997	0.630599976	0.474299997
4	-0.324299991	-2.16000006E-02	-0.538500011
5	-6.23000003E-02	0.415399998	0.175699994
6	-8.39999989E-02	-0.127900004	-8.10000021E-03
7	-0.460200012	-0.107199997	0.550000012
8	-0.196700007	-8.89999978E-03	-5.10000018E-03
9	-1.62000004E-02	0.639599979	0.218400002
10	-1.32999998E-02	0.154300004	-0.199399993
11	-0.666999996	-0.354099989	-0.381599993
12	-9.62999985E-02	5.33000007E-02	7.16999993E-02
13	0.282700002	8.57999995E-02	-8.70999992E-02
14	0.153400004	1.39999995E-03	0.386500001
15	0.265899986	0.366400003	2.36000009E-02
16	3.35000008E-02	-0.371499985	0.160799995

Ground energy
-686.905090
Excitation energy
6.79430008
6.87050009
6.91039991
7.10680008
7.41249990
7.52199984
7.61590004

7.83500004
7.86089993
7.87069988
7.94339991
7.98769999
8.13319969
8.19349957
8.30249977
8.33290005

E-Beta
Number of atoms: 24

1	3.29334	-0.30728	-1.45475
8	3.49309	0.08436	-0.59242
6	2.60553	-0.47467	0.35809
1	2.68158	0.13224	1.25996
1	2.87555	-1.51009	0.6072
6	1.16999	-0.47125	-0.14604
1	1.1353	-0.93223	-1.14378
6	0.57762	0.92999	-0.2505
1	1.10527	1.46024	-1.05294
8	0.76327	1.60734	0.98664
1	0.73423	2.5535	0.7908
6	-0.91896	0.88743	-0.5746
1	-1.07519	0.49156	-1.5813
8	-1.44868	2.20154	-0.56947
1	-1.72657	2.40182	0.33529
6	-1.61033	-0.02595	0.42378
1	-1.52245	0.4162	1.42657
8	-2.97058	-0.17514	0.04276
1	-3.42737	-0.65737	0.74778
6	-0.9218	-1.39673	0.43553
8	0.42425	-1.28259	0.76052
1	-1.36	-2.03855	1.21379
8	-1.03113	-1.99898	-0.83359
1	-1.96777	-2.19518	-0.98172

Electric moments

0	0	-2.61129999	-1.01030004	0.853799999
0	1	4.69999984E-02	0.109999999	-0.129700005
0	2	-9.73000005E-02	8.42000023E-02	5.68999983E-02
0	3	-8.70999992E-02	4.43000011E-02	-4.85999994E-02
0	4	0.116200000	-9.01999995E-02	-0.105200000
0	5	0.185399994	-0.100699998	0.140499994
0	6	-0.160300002	-6.23999983E-02	0.251100004
0	7	1.43999998E-02	-0.223299995	7.40000010E-02
0	8	0.386700004	0.150600001	-6.97000027E-02
0	9	-0.292499989	-0.273799986	0.245700002
0	10	0.123999998	-7.32000023E-02	-0.161899999
0	11	8.60000029E-02	9.60000008E-02	0.141000003
0	12	8.16000029E-02	-0.105099998	-9.67999995E-02
0	13	-5.55999987E-02	2.42999997E-02	0.184599996
0	14	0.139300004	-0.275799990	-7.47999996E-02

0	15	1.72000006E-02	-7.84000009E-02	0.124100000
0	16	-2.00999994E-02	0.209900007	4.19999985E-03
1	1	4.05700016	1.52980006	-2.90319991
2	2	-2.00990009	-5.27549982	-0.439399987
3	3	-2.96600008	-0.426200002	4.31689978
4	4	0.358200014	1.13220000	0.804400027
5	5	0.816999972	-1.84759998	-1.36039996
6	6	-1.46159995	1.17429996	1.01820004
7	7	-0.845399976	-3.11540008	-0.549600005
8	8	2.43400002	1.29709995	-2.47679996
9	9	-0.465700001	-2.70560002	-0.108900003
10	10	1.65090001	1.71120000	-1.71889997
11	11	0.883199990	2.98070002	0.459500015
12	12	-4.06739998	-1.79929996	2.95910001
13	13	1.18589997	-2.67750001	-2.50830007
14	14	-2.38470006	-0.635800004	2.41240001
15	15	-1.00549996	-0.523800015	1.53849995
16	16	2.75359988	0.766900003	-1.12189996

Magnetic moments

1	6.76999986E-02	-0.102600001	0.483900011
2	-0.329899997	4.98000011E-02	1.37000000E-02
3	-0.300300002	-0.455100000	-0.410299987
4	0.208000004	0.230299994	-0.331600010
5	0.375000000	-0.294400007	0.263399988
6	0.259200007	-0.294600010	0.564899981
7	-0.254500002	0.126499996	0.290199995
8	-8.57999995E-02	-0.173199996	0.597700000
9	-0.122800000	-0.222100005	-0.833100021
10	0.134499997	-0.136500001	2.81000007E-02
11	3.07999998E-02	-9.12000015E-02	0.112300001
12	0.335500002	-0.397899985	9.13000032E-02
13	-4.82000001E-02	0.150700003	6.71000034E-02
14	0.302500010	-0.286199987	0.314799994
15	-0.376100004	0.278400004	0.382299989
16	5.88999987E-02	9.31999981E-02	0.331600010

Ground energy

-686.910461

Excitation energy

6.46339989
6.76270008
6.82200003
7.19649982
7.31580019
7.46129990
7.56090021
7.58419991
7.63430023
7.82800007
7.86180019
7.94780016
8.00520039
8.16269970

8.20219994
8.25409985

F-Beta
Number of atoms: 24

1	2.73308	-0.99348	-0.69824
8	3.19801	-0.14313	-0.6429
6	2.64586	0.57901	0.42247
1	2.91955	1.62911	0.28591
1	3.06994	0.25313	1.38937
6	1.12555	0.51713	0.56284
1	0.85912	0.98403	1.52062
6	0.53436	-0.8928	0.5719
1	0.84983	-1.39506	1.49913
8	0.98915	-1.62918	-0.55235
1	0.48295	-2.45824	-0.55349
6	-0.9918	-0.85369	0.54563
1	-1.35465	-0.44413	1.49955
8	-1.39343	-2.20977	0.39258
1	-2.35734	-2.26249	0.44375
6	-1.49214	0.01864	-0.59469
1	-1.19953	-0.43447	-1.54421
8	-2.90074	0.16537	-0.59911
1	-3.23021	0.03077	0.30003
6	-0.82561	1.39227	-0.48699
8	0.57214	1.28937	-0.50403
1	-1.08149	2.00454	-1.35764
8	-1.30056	1.98073	0.70137
1	-0.98515	2.89587	0.72389

Electric moments

0	0	-2.99710011	-0.151899993	3.61299992
0	1	0.149700001	1.72000006E-02	0.100400001
0	2	-8.60999972E-02	9.14999992E-02	0.194199994
0	3	3.42999995E-02	0.132900000	9.49999969E-03
0	4	-7.97000006E-02	0.209199995	-4.10000011E-02
0	5	0.149599999	-8.63000005E-02	-0.158800006
0	6	-0.122000001	-4.96000014E-02	-2.67999992E-02
0	7	0.167999998	-0.223000005	0.363599986
0	8	-1.89999994E-02	-8.10000002E-02	4.30999994E-02
0	9	-1.37000000E-02	-0.101300001	0.268999994
0	10	8.03000033E-02	-6.15000017E-02	0.197799996
0	11	-0.186000004	5.27999997E-02	-0.222200006
0	12	3.40999998E-02	0.134399995	-9.22999978E-02
0	13	6.06999993E-02	0.102100000	0.205599993
0	14	-6.98999986E-02	-9.30000003E-03	3.81000005E-02
0	15	0.199300006	-7.93000013E-02	0.118400000
0	16	-1.57999992E-02	3.22999991E-02	0.103200004
1	1	2.83620000	3.49880004	-0.186800003
2	2	-1.33879995	1.03199995	-0.656199992
3	3	1.33539999	1.86409998	0.664200008
4	4	-2.27690005	-5.80249977	1.58800006
5	5	-0.432999998	3.73469996	1.51390004

6 6 -1.10450006 -1.14800000 1.39859998
7 7 0.227799997 -0.647499979 1.07889998
8 8 7.61759996 1.56750000 0.830699980
9 9 1.71039999 -1.92600000 1.01010001
10 10 0.319700003 -1.46089995 2.62680006
11 11 -0.707300007 -1.97700000 1.38600004
12 12 -0.907800019 -0.231900007 0.819899976
13 13 0.229599997 1.14489996 0.294200003
14 14 -0.287800014 1.06710005 1.28020000
15 15 0.169100001 -3.74489999 0.961600006
16 16 3.23920012 3.66969991 0.808300018

Magnetic moments

1 0.180099994 -9.60000046E-03 0.567799985
2 -0.223499998 -9.35999975E-02 -8.47999975E-02
3 -0.497200012 0.146699995 0.461199999
4 7.41999969E-02 -2.71000005E-02 -0.174400002
5 0.370999992 -5.35999984E-02 -0.132400006
6 0.149399996 -0.454499990 -0.320699990
7 -9.20000020E-03 0.363599986 -0.255400002
8 -4.80000023E-03 0.225299999 -5.90000004E-02
9 0.627300024 0.676599979 -0.195999995
10 -0.377700001 0.503799975 -0.231199995
11 6.93000033E-02 0.302399993 -7.51999989E-02
12 0.247999996 0.515600026 6.84999973E-02
13 0.515200019 -0.541400015 0.251100004
14 -0.281100005 -0.454600006 0.411300004
15 9.64000002E-02 -0.244599998 0.139100000
16 0.229599997 3.22000012E-02 -3.24000008E-02

Ground energy

-686.918457

Excitation energy

6.51959991
6.91169977
6.99760008
7.09110022
7.45270014
7.49139977
7.56790018
7.61129999
7.70340014
7.82730007
7.87190008
7.89639997
7.97279978
8.03190041
8.12500000
8.16079998

G-Beta

Number of atoms: 24

1 3.11323 -1.33575 -0.36872
8 3.58096 -0.49696 -0.2636

6	2.62442	0.50219	0.03635
1	2.87388	1.39391	-0.54893
1	2.6697	0.77624	1.09648
6	1.20953	0.07238	-0.29615
1	1.2191	-0.42431	-1.27729
6	0.21834	1.22718	-0.36447
1	0.47765	1.87457	-1.20655
8	0.31812	2.06228	0.78315
1	-0.01892	1.5729	1.54791
6	-1.20824	0.70851	-0.57355
1	-1.2428	0.21016	-1.54851
8	-2.13382	1.76221	-0.6584
1	-2.18134	2.18668	0.21105
6	-1.5565	-0.33503	0.49362
1	-1.57854	0.13676	1.4843
8	-2.82613	-0.909	0.33012
1	-2.84041	-1.31665	-0.5496
6	-0.47207	-1.41954	0.51939
8	0.81426	-0.88138	0.70092
1	-0.62628	-2.08859	1.37207
8	-0.59651	-2.11611	-0.69653
1	-0.01333	-2.88841	-0.66868

Electric moments

0	0	-0.263000011	-2.50720000	0.198400006
0	1	-0.115300000	-7.15999976E-02	-0.204500005
0	2	-3.40000005E-03	-7.45000020E-02	-6.58999979E-02
0	3	8.32000002E-02	-4.03999984E-02	-3.88999991E-02
0	4	-6.03000000E-02	4.98999991E-02	-4.69999984E-02
0	5	0.177599996	-7.81999975E-02	-3.55000012E-02
0	6	0.178299993	0.258399993	-0.293399990
0	7	1.97000001E-02	0.186600000	-0.103699997
0	8	-0.163599998	-4.45999987E-02	0.132499993
0	9	-0.214100003	-5.73999994E-02	-3.90999988E-02
0	10	2.96999998E-02	0.224399999	-0.150900006
0	11	-7.45000020E-02	-0.175500005	0.158099994
0	12	3.80000006E-03	-3.33999991E-02	0.275099993
0	13	-4.74999994E-02	0.267100006	-9.70000029E-03
0	14	-2.63999999E-02	0.125400007	-0.112700000
0	15	5.95999993E-02	0.176200002	1.41000003E-02
0	16	-0.117600001	7.19999988E-03	-0.125200003
1	1	3.92000005E-02	3.43039989	0.790400028
2	2	0.738799989	-3.41370010	-4.29320002
3	3	-0.134800002	1.39279997	-0.983799994
4	4	-2.86999997E-02	-2.65580010	-1.99199998
5	5	0.650500000	0.297899991	2.71259999
6	6	-1.33130002	1.15709996	-0.206900001
7	7	0.677500010	-1.15579998	2.64770007
8	8	-0.705299973	0.806999981	0.753700018
9	9	-2.65660000	2.63190007	1.12860000
10	10	-0.330599993	-3.76270008	-0.754400015
11	11	-1.36469996	-0.123800002	-1.03670001
12	12	3.08890009	-1.12609994	-1.46519995

13 13 -9.79000032E-02 0.393599987 0.482400000
14 14 4.46560001 -0.947899997 -0.217700005
15 15 1.36210001 -1.15590000 1.75610006
16 16 -0.883700013 -3.29520011 -1.06340003

Magnetic moments

1 -0.374799997 -0.358399987 0.147400007
2 -0.201000005 -0.298799992 0.174400002
3 -0.137999997 -0.619199991 -0.233400002
4 -0.154899999 -0.279799998 -0.432099998
5 0.166299999 -2.98999995E-02 0.477899998
6 0.201900005 -8.34999979E-02 0.524100006
7 -0.130199999 0.320100009 0.649699986
8 0.254099995 6.44999966E-02 -0.142800003
9 0.555700004 -1.67999994E-02 0.342700005
10 -2.52999999E-02 0.319299996 -6.49000034E-02
11 -0.693400025 -0.322299987 0.378399998
12 0.314799994 0.208600000 -8.89999978E-03
13 -0.173199996 6.47000000E-02 -0.214000002
14 0.153500006 -0.626999974 3.35000008E-02
15 -0.143500000 0.259099990 -0.188899994
16 0.231800005 2.05000006E-02 0.156200007

Ground energy

-686.914124

Excitation energy

6.73050022
6.85540009
6.93400002
7.13159990
7.35790014
7.53109980
7.53520012
7.70030022
7.77610016
7.85370016
7.96210003
8.04539967
8.08139992
8.17899990
8.19610023
8.25689983

H-Beta

Number of atoms: 24

1 -2.80894 1.39213 -1.15073
8 -3.30209 0.96183 -0.43949
6 -2.65164 -0.24647 -0.1103
1 -3.0846 -1.09817 -0.65734
1 -2.81481 -0.41971 0.95514
6 -1.16024 -0.22197 -0.39663
1 -0.98465 -0.19508 -1.48292
6 -0.41017 -1.4196 0.19711
1 -0.746 -2.34392 -0.27995

8	-0.68673	-1.55943	1.57604
1	-0.44834	-0.72232	2.00363
6	1.09105	-1.26639	-0.07017
1	1.62162	-2.0621	0.46752
8	1.27374	-1.38675	-1.46986
1	2.20701	-1.58728	-1.62446
6	1.59637	0.08907	0.41337
1	1.50285	0.15796	1.50807
8	2.94304	0.214	0.01437
1	3.20489	1.13218	0.1823
6	0.72615	1.18709	-0.17509
8	-0.62403	0.97212	0.18769
1	0.8113	1.18517	-1.2731
8	1.14241	2.39773	0.37347
1	0.42	3.03465	0.2642

Electric moments

0	0	1.83130002	0.671100020	-1.37839997
0	1	-3.07000000E-02	5.48000000E-02	-7.22000003E-02
0	2	0.119400002	0.101099998	0.171000004
0	3	9.30999964E-02	2.79999990E-03	4.45999987E-02
0	4	-0.268700004	3.92999984E-02	7.22000003E-02
0	5	9.76999998E-02	-0.179199994	-0.170699999
0	6	-7.94000030E-02	0.221100003	-0.368099988
0	7	-4.28000018E-02	8.00000038E-03	-0.169300005
0	8	6.36000037E-02	-8.85000005E-02	-4.16000001E-02
0	9	-2.15000007E-02	-5.70000000E-02	-0.187800005
0	10	-2.97999997E-02	-0.105700001	-2.86999997E-02
0	11	5.70000010E-03	-0.265500009	-0.112899996
0	12	-1.98999997E-02	0.139200002	3.88000011E-02
0	13	7.66000003E-02	8.95999968E-02	0.117899999
0	14	-0.149200007	5.16000018E-02	0.190300003
0	15	-0.134800002	-0.259600013	0.404199988
0	16	-0.169200003	-0.199200004	4.60000001E-02
1	1	1.43859994	-3.52259994	2.68549991
2	2	2.35290003	-5.20069981	1.92429996
3	3	-1.92040002	1.63320005	0.140300006
4	4	0.456000000	1.22239995	-5.02899981
5	5	-1.56930006	0.766799986	-1.75469995
6	6	1.58270001	-0.557200015	1.98800004
7	7	1.96459997	1.59809995	-6.62470007
8	8	-1.84060001	2.39569998	0.754599988
9	9	2.27430010	-2.99460006	1.48300004
10	10	1.14590001	0.802800000	-0.139100000
11	11	2.06150007	3.20740008	-0.967800021
12	12	-1.56389999	0.604099989	-4.04239988
13	13	1.29380000	-1.54869998	-0.823300004
14	14	3.25959992	0.857800007	0.114500001
15	15	1.23080003	0.258599997	-4.86880016
16	16	1.91199994	2.10920000	1.12329996

Magnetic moments

1	0.646799982	0.284500003	-0.309899986
2	0.170200005	0.178700000	0.219699994

3 -0.497799993 0.128600001 -4.90000006E-03
4 3.55000012E-02 0.483599991 0.265899986
5 0.361999989 0.112000003 0.129500002
6 0.422300011 -0.367000014 -0.116200000
7 0.389400005 0.564599991 0.172399998
8 0.225400001 -0.667699993 0.252099991
9 0.297199994 -0.276199996 0.255100012
10 -0.263500005 -0.174199998 -0.486499995
11 -0.157600001 0.352499992 -7.09000006E-02
12 -9.39999968E-02 -7.86999986E-02 0.445100009
13 -0.326599985 0.246900007 0.197099999
14 -0.502799988 -0.163399994 -0.230100006
15 -5.04000001E-02 0.317000002 6.98999986E-02
16 -2.71000005E-02 0.109099999 -2.09999997E-02

Ground energy
-686.917236

Excitation energies
6.74109983
6.86040020
7.12519979
7.24580002
7.25589991
7.52199984
7.69939995
7.78859997
7.87410021
8.00479984
8.02280045
8.12860012
8.17889977
8.22910023
8.32590008
8.35179996

S2. Composition of the essential eigenvectors of the covariance matrix

Composition of the first two eigenvectors (number of atoms are reported at the end of this paragraph) of the covariance matrix for:

D-glucose-Alpha

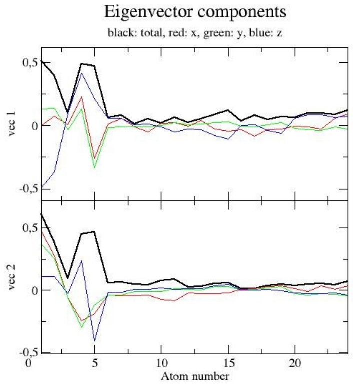

D-glucose-Beta

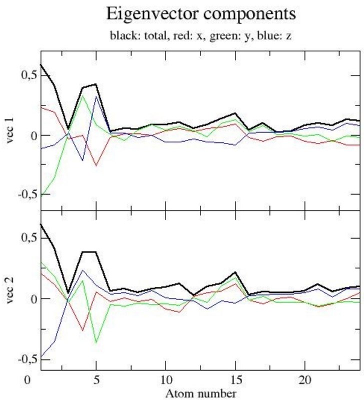

D-galactose-Alpha

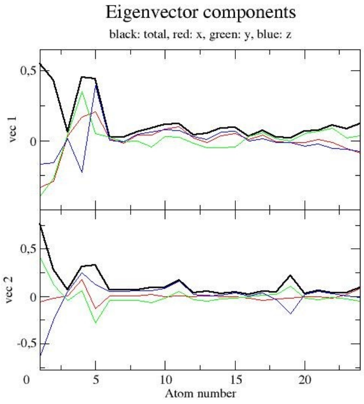

D-galactose-Beta

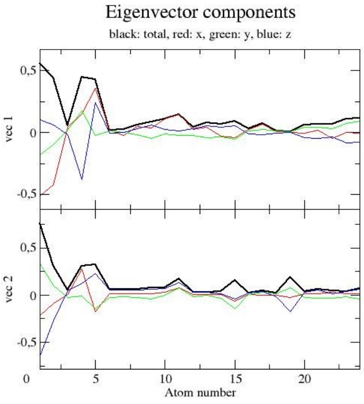

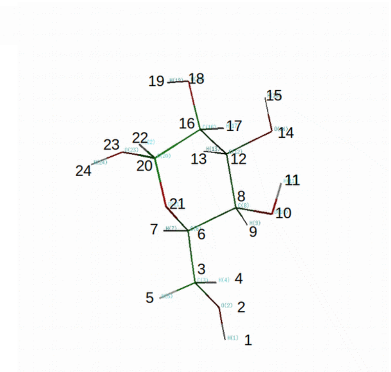

S3. Location of the Representative Conformations on the Essential plane

D-Glucose-Alpha

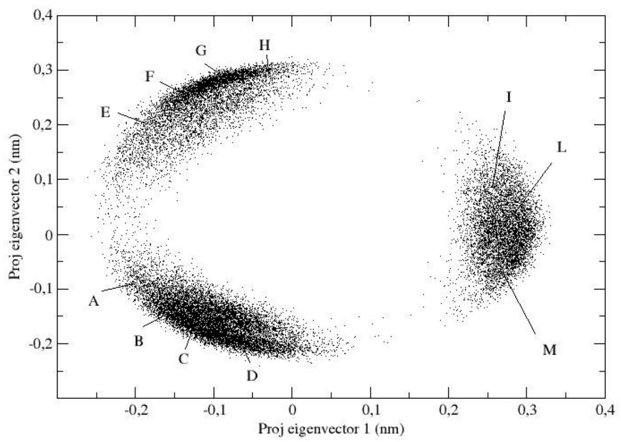

D-Glucose-Beta

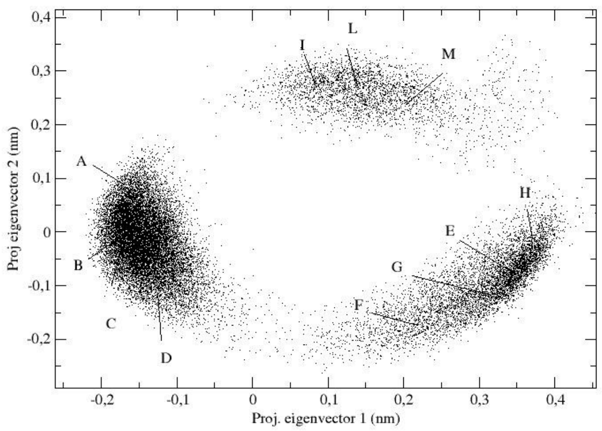

D-Galactose-Alpha

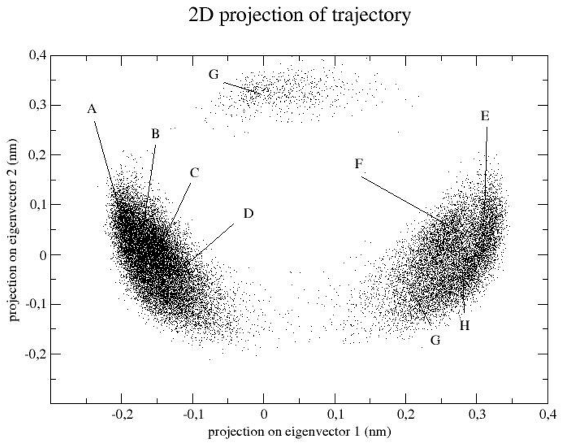

D-Galactose-Beta

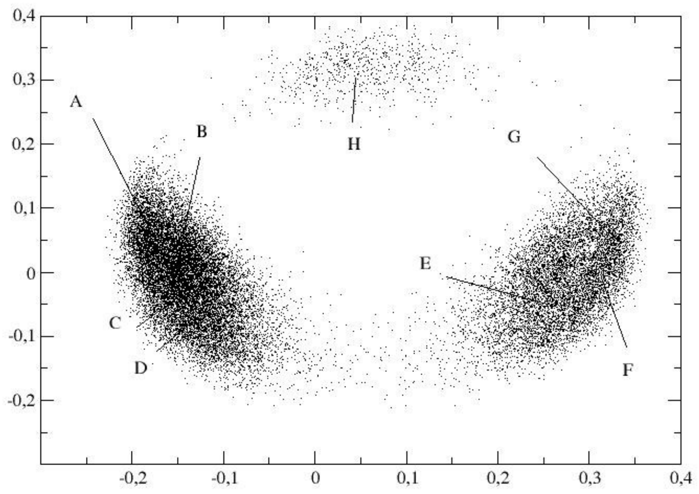

S4. Spectra of the GG and GT rotamers for Alpha and Beta D-glucose

Spectra of the GG and GT rotamers for Alpha and Beta D-glucose

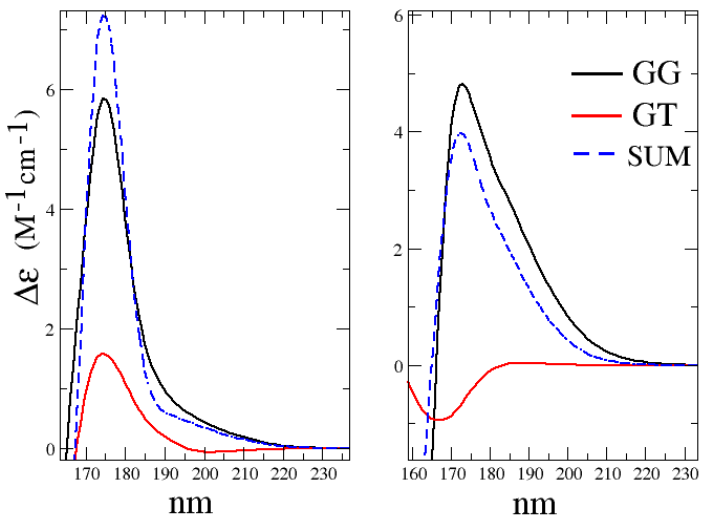

S5. Eigenvalues of the D-Galactose covariance matrix

Spectra of the eigenvalues from the diagonalization of the covariance matrix for Alpha (black) and Beta (red) D-galactose.

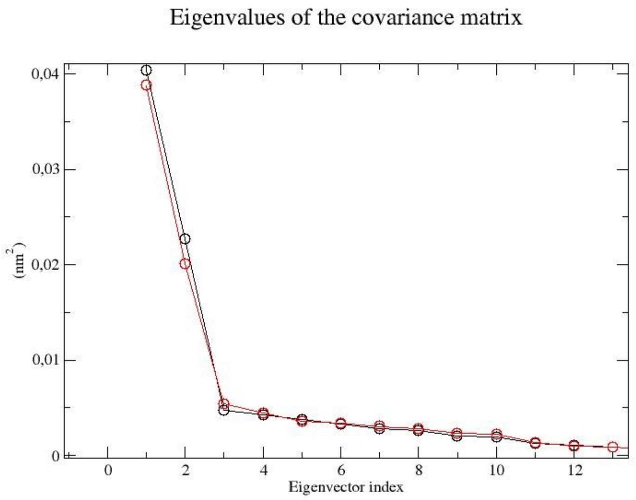

Supplement: Supplementary file 1 [file molecules-28-03591-s001.zip › molecules-2335563-supplementary.pdf]
